# Global disease burden attributed to unsafe sex in 204 countries and territories from 1990 to 2019: results from the Global Burden of Disease Study 2019

**DOI:** 10.1038/s41598-023-40001-2

**Published:** 2023-08-09

**Authors:** Pei Qiu, Hairong He, Yuting Zhao, Zejian Yang, Shouyu Li, Peng Ni, Yujie Guo, Chao Ji, Chenchen Zhang, Huimin Zhang, Can Zhou, Bo Wang

**Affiliations:** 1 Department of Breast Surgery, The First Affiliated Hospital of Xi’an Jiaotong University, 277 Yanta Western Rd., Xi’an, 710061 Shaan’xi China; 2 School of Medicine, Xi’an Jiaotong University, Xi’an, Shaanxi China; 3 Clinical Research Center, The First Affiliated Hospital of Xi’an Jiaotong University, Xi’an, Shaanxi China; 4 Department of Clinical Laboratory, The 940th Hospital of Joint Logistics Support Force of Chinese People’s Liberation Army, Lanzhou, China; 5 Center for Translational Medicine, The First Affiliated Hospital of Xi’an Jiaotong University, 277 Yanta Western Rd., Xi’an, 710061 Shaan’xi China; 6 Key Laboratory for Tumor Precision Medicine of Shaanxi Province, Xi’an, China

**Keywords:** Health care, Risk factors

## Abstract

Unsafe sex has become a public safety problem that endangers society, and research on deaths and disability-adjusted life years (DALYs) related to unsafe sex is valuable for global policy-making. We aimed to estimate the deaths and DALYs attributable to unsafe sex by country, gender, age group, and sociodemographic status from 1990 to 2019. We extracted data on disease burden from the Global Disease Burden 2019 (GBD 2019) database for unsafe sex, including deaths, DALYs and age-standardized rates (ASRs). Comparative analyses were performed on data about deaths, DALYs and the responding ASRs attributable to unsafe sex in different countries and regions using the Social Demographic Index (SDI). The global age-standardized mortality rate (ASMR) and age-standardized DALY rate (ASDR) attributable to unsafe sex were 11.98 (95% uncertainty intervals (UI): 10.97–13.52) per 100,000 people and 570.78 (95% UI: 510.24–658.10) per 100,000 people, respectively. Both the ASMRs and ASDRs were the highest in southern sub-Saharan Africa and lowest in Australasia and decreased with increasing SDI levels. About unsafe-sex-related disease, HIV/AIDS has the highest ASMR [8.48 (95% UI: 7.62–9.95)/100,000 people] and ASDR [447.44 (95% UI: 394.82–533.10)/100,000 people], followed by Cervical cancer [ASMR: 3.40 (95% UI: 2.90–3.81)/100,000 people and ASDR: 107.2 (95% UI: 90.52–119.43)/100,000 people] and sexually transmitted infections excluding HIV [ASMR: 0.10 (95% UI: 0.08–0.11)/100,000 people and ASDR: 16.14 (95% UI: 10.51–25.83)/100,000 people]. The death and DALY burden caused by these three diseases were more serious in the over 75 years old age group. The 40–44 age group for men and the 35–39 age group for women had the highest population of unsafe sex-related deaths and DALYs, respectively. In addition, the burden of unsafe sex in women was more serious than those in men. Unsafe sex is an important risk factor for global disease burden and a leading cause of substantial health loss. We found that the risk of ASMRs and ASDRs attributable to unsafe sex had negative correlation with SDI levels. These results demonstrate that the need for revised policies that focus on efforts to reduce overall unsafe sex worldwide.

## Introduction

Unsafe sex, which includes unprotected sex^1^, sex influenced by alcohol or drugs^2^, and sex exchanged for money or drugs^3^, refers to sexual behavior performed without precautions for contraception or avoiding acquiring or spreading sexually transmitted diseases or while in an unhealthy mental state^4^. The consequently serious consequences originating from unsafe sex could be ascribed to three aspects: physical damage, psychological damage and unwanted pregnancy. Physical damage is the most common occurrence of unsafe sex in a variety of illness situations, such as the infection of human papillomavirus (HPV) during unprotected sexual intercourse and sexually transmitted diseases (STDs) though consistent condomless use^5,6^. Psychological damage is the negative psychological outcome that appears after unsafe sex, such as perpetrator, situational blame and societal blame, negative thoughts about the self and even the world^7,8^. Unintended pregnancy seems to be a frequent accident that affects reproductive health^9–11^, and it is one of the major negative consequences of unsafe sex^12^.

Meanwhile, unsafe sex is associated with HPV-dependent cancers^13–15^. Annually, more than 35,900 cancers contribute to HPV infections annually in the United States^16^. These HPV-associated cancers, defined as invasive carcinomas lying at specific anatomic locations and comprising specific cell types of HPV DNA, comprise cervical cancer and vaginal, vulvar, oropharyngeal, anal, and penile neoplasms^17,18^. It is estimated that HPV infections may induce as many as 50–80% of penile neoplasms^19^. Therefore, unsafe sex, which is self-evident in various STDs, has evolved into a public safety problem that endangers every person, every country and even all humanity, despite current vaccines reducing most HPV-associated cancers^13^. Further knowledge about the enormous influence of unsafe sex on public health is necessary to better allocate limited health resources worldwide, which is valuable for reducing the burden attributable to unsafe sex and for policy-making regarding health resource planning.

Thus far, several regional and national studies on public health and unsafe sex have been conducted, and the findings of these multifarious studies from entirely different parts of the world present a recapitulatory map. Epidemiological studies from Kenya^20^, Cameroon^21^, South Africa^22^ and Denish^23^ demonstrate a significant burden of unsafe sex with associated STDs and HPV-associated cancers. However, due to the lack of specific studies, there has been no detailed assessment of the unsafe sex burden at a global level. To further explore and identify the subsequent disease burden caused by unsafe sex more succinctly and intuitively, this study was conducted based on GBD data from 1990 to 2019. The current research would provide important epidemiological clues for the prevention of unsafe sex and subsequent diseases, and can help establish policies on health resources worldwide.

## Methods

### Study data sources

Data sources for unsafe sex burdens were obtained from an online data source tool, the Global Health Data Exchange (GHDx) query tool (https://ghdx.healthdata.org/gbd-results-tool). We extracted data according to GBD’s operational guidelines. GBD 2019 iteration was the latest and superseded the results of prior GBD studies. We obtained data on mortality, DALY and age-standardized rate (ASR) of unsafe sex from 1990 to 2019 according to sex, 21 regions, and 204 countries/territories. The sociodemographic index (SDI) was defined as the geometric average of total fertility, per capita income, and average years of education, and the SDI ranged from 0 to 1^24,25^. SDI was a summary metric of overall development, and was used to describe the differences in the burden of disease attributable to risk factors across the spectrum of sociodemographic development. The 204 countries/territories were divided into 5 categories based on the SDI in 2019, including low, low-middle, middle, high-middle, and high SDI regions. This study was approved by the Ethics Committee of the First Affiliated Hospital of Xi'an Jiaotong University.

The main data sources used to estimate the attributable burden due to unsafe sex were Joint United Nations Programme on HIV/AIDS and Government epidemiological surveillance records^26^. The outcomes associated with unsafe sex was estimated for GBD include HIV, cervical cancer, and all STDs except for newborns with vertical transmission. Based on evidence in the literature, the GBD study assumed that 100% of cervical cancer and STDs could be attributed to unsafe sex. By modeling the proportion of HIV incidence occurring through sexual transmission, the attributable burden due to unsafe sex for HIV could be estimated. Three DisMod-MR (disease model—Bayesian meta-regression) models were used, and the results were adjusted to sum to one^26^. As high as 1000 draws were taken from the posterior distribution and sorted. The 95% uncertainty intervals (UIs) were calculated by selecting the 25th draw for the lower bound and the 975th draw for the upper bound of uncertainty. The mean was taken across the draws to calculate the point estimate^27^.

### Definitions

The theoretical minimum level for unsafe sex was defined as the absence of disease transmission through sexual contact. Years lived with disability (YLDs) were calculated as the prevalence of each sequela times a corresponding GBD disability weight derived from a population survey of > 60,000 respondents^28,29^. Years of life lost (YLLs) due to premature mortality were calculated using the number of deaths and standard global life expectancy at the age of death in years. DALY was used to estimate the global cancer burden, and can be calculated by combining YLL and YLD measures of disease burden.

In GBD 2019, the causes of disease and injuries are categorized into four mutually exclusive levels. The three cause groups at level 1 are communicable, maternal, neonatal, and nutritional diseases; noncommunicable diseases; and injuries. There are 22 categories of diseases and injuries in level 2 as the subgroups of level 1, such as HIV/AIDS and sexually transmitted infections in communicable, maternal, neonatal, and nutritional diseases. Level 3 represents more detailed causes within the level 2 categories, such as HIV/AIDS within HIV/AIDS and sexually transmitted infections. Level 4 includes some subcauses of level 3 causes, such as HIV/AIDS—drug-susceptible tuberculosis in HIV/AIDS.

The population was divided into 17 age groups every 5 years, which were younger than 20 age group, 20–24 age group, 25–29 age group, 30–34 age group, 35–39 age group, 40–44 age group, 45–49 age group, 50–54 age group, 55–59 age group, 60–64 age group, 65–69 age group, 70–74 age group, 75–79 age group, 80–84 age group, 85–89 age group, 90–94 age group and 95 and older age group. When analyzing differences in the number of deaths and DALYs at different SDI levels in different age groups, to simplify the grouping, the population was grouped into four groups every 25 years: younger than 25 years, 25–49 years, 50–74 years and 75 and older years.

### Statistical methods

ASRs, mortality and DALYs were calculated to demonstrate the burden of unsafe sex. DALYs refers to the years lived with disability and years of life lost per 100,000 people. ASRs (age-standardized death/DALY rate) (per 100,000 people), were calculated using the following formula:

$${\text{ASR}} = \frac{{\sum\nolimits_{i = 1}^{A} {a_{i} w_{i} } }}{{\sum\nolimits_{i = 1}^{A} {w_{i} } }} \times 100{,}000,$$

where a_i_ represents the age-specific rate in the ith age group, w represents the number of people (or the weight) in the same ith age group from among the selected reference standard population, and A represents the number of age groups. Pearson correlation analysis was used to analyze the correlation between SDI and ASR. Bilateral *P* < 0.05 was considered statistically significant. All calculations were performed using GraphPad Prism 8 and R software (version 4.0.2).

### Ethics approval and consent to participate

This study was approved by the Ethics Committee of the First Affiliated Hospital of Xi'an Jiaotong University. The GBD database does not use directly patients’ records, so there is no need for informed consent from the patients. The waiver of informed consent was approved by the Institutional Review Board (IRB). All methods were carried out in accordance with relevant guidelines and regulations.

## Results

### Global burden attributable to unsafe sex

**Figure 1:**
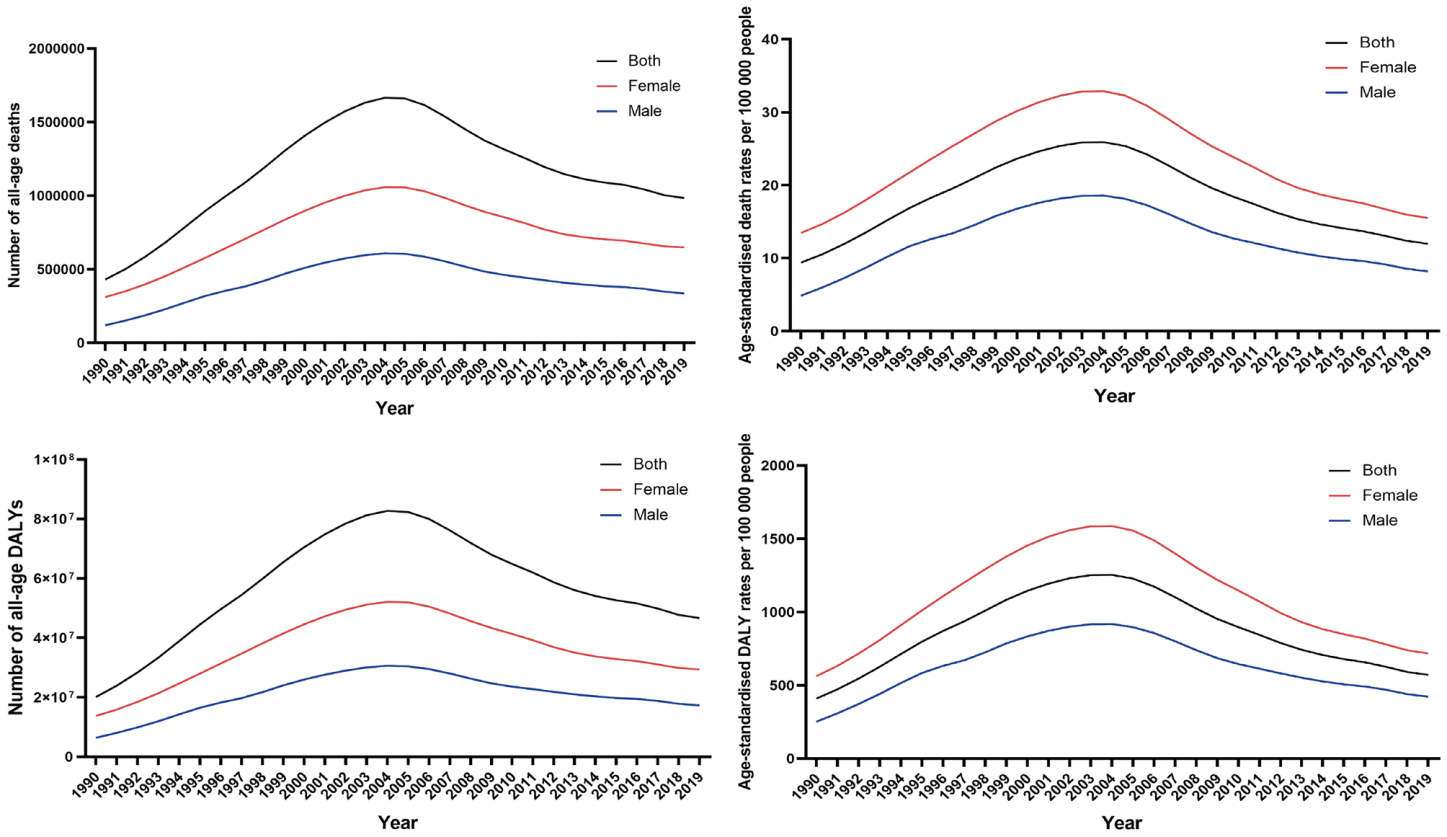
The numbers and rates of deaths and DALYs attributable to unsafe sex globally from 1990 to 2019.

From 1990 to 2019, the number of global deaths and DALYs attributed to unsafe sex tended to increase gradually (Fig. 1), with a rapid increase in the first 14 years and then a dramatic decrease in the last 14 years. The number of global deaths attributable to unsafe sex increased rapidly from 430,000 (95% UI: 360,000–530,000) cases in 1990, peaked at 1.7 million (95% UI: 1.3–2) cases in 2004, and then slowly declined to 1 million (95% UI: 0.9–1.1) cases in 2019. Similarly, the number of global DALYs attributable to unsafe sex rose from 20 million (95% UI: 16.4–25.6) cases in 1990 to 83 million (95% UI: 64.7–103.1) cases in 2004 and then decreased to 47 million (95% UI: 41.9–53.5) cases in 2019.

From 1990 to 2019, both the ASMR and ASDR showed decreasing trends. The global ASMR increased from 9.38/100,000 people in 1990 to 11.98/100,000 people in 2019, with an absolute increase of 2.6%, while the ASDR increased from 409.67/100,000 people in 1990 to 570.78/100,000 people in 2019, with an absolute increase of 169.11/100,000 people. The ASMR and ASDR achieved congruence in the trend, which demonstrated an upward trajectory between 1990 and 2004, followed by a slow decline between 2005 and 2019.

### Burden attributable to unsafe sex in various regions

(Table 1) Regionally, in 2019, both the ASMR and ASDR, 237.92 (95% UI: 212.64–279.30)/100,000 people and 11,711.17 (95% UI: 10,265.58–14,089.39)/100,000 people, respectively, were the highest in southern sub-Saharan Africa and the lowest in Australasia [1.38 (95% UI: 1.22–1.50)/100,000 people and 57.37 (95% UI: 49.74–66.45)/100,000 people in ASDR] attributable to unsafe sex. Southern Sub-Saharan Africa was the fastest growing region in deaths and DALYs from 1990 to 2019, with increases of 542.00% (95% UI: 283.30–855.70%) in deaths and 538.35% (95% UI: 284.94–820.69%) in DALYs. High-income North American regions demonstrated the largest steps in reducing deaths and delays from 1990 to 2019, with a reduction of 67.30% (95% UI: 64.76–69.29%) in deaths and 67.29% (95% UI: 62.64–70.74%) in DALYs.

**Table 1:** Global, regional and national ASRs of deaths and DALYs attributable to unsafe sex in 2019 and percentage changes from 1990 to 2019. The bold text means 21 GBD regions.

	Deaths		DALYs	
	2019 age-standardized rate per 100, 000 people	Percentage change in age-standardized rate, 1990–2019	2019 age-standardized rate per 100, 000 people	Percentage change in age-standardized rate, 1990–2019
**Global**	**11.98 (10.97, 13.52)**	**27.64% (13.89%, 44.60%)**	**570.78 (510.24, 658.10)**	**39.33% (25.14%, 58.37%)**
Low-SDI quintile	34.33 (31.13, 38.55)	− 29.40% (− 44.35%, − 5.58%)	1579.77 (1410.49, 1829.90)	− 31.68% (− 45.33%, − 10.23%)
Low-middle-SDI quintile	16.95 (15.59, 18.95)	61.57% (34.32%, 91.24%)	776.91 (700.54, 883.35)	75.25% (44.20%, 107.83%)
Middle-SDI quintile	12.22 (11.03, 13.95)	116.28% (75.02%, 146.32%)	567.12 (508.55, 660.33)	183.31% (134.83%, 222.39%)
High-middle-SDI quintile	4.35 (3.82, 4.75)	− 9.65% (− 24.63%, − 0.70%)	184.40 (164.50, 202.50)	5.04% (− 11.12%, 14.07%)
High-SDI quintile	2.24 (2.06, 2.36)	− 60.89% (− 63.23%, − 58.52%)	96.88 (85.99, 110.93)	− 61.46% (− 64.93%, − 57.23%)
**Central Sub-Saharan Africa**	**49.85 (42.17, 59.17)**	− **18.01% (**− **37.84%, 8.05%)**	**2207.76 (1850.96, 2672.43)**	− **21.23% (**− **39.70%, 3.82%)**
Angola	83.76 (61.50, 114.63)	400.07% (226.02%, 659.97%)	3882.27 (2778.72, 5417.95)	532.69% (310.70%, 863.89%)
Central African Republic	150.41 (124.30, 190.61)	81.81% (17.41%, 155.97%)	6834.70 (5559.11, 8946.15)	64.37% (7.59%, 127.90%)
Congo	94.41 (80.12, 112.77)	− 37.57% (− 57.54%, − 7.58%)	4336.97 (3638.97, 5222.58)	− 38.92% (− 58.17%, − 10.95%)
Democratic Republic of the Congo	26.11 (20.29, 32.83)	− 60.95% (− 73.15%, − 40.53%)	1063.49 (849.23, 1348.81)	− 65.75% (− 76.13%, − 47.48%)
Equatorial Guinea	197.03 (129.29, 305.77)	797.25% (421.16%, 1436.93%)	9443.61 (6082.09, 14,869.52)	1000.00% (514.75%, 1810.18%)
Gabon	76.37 (57.05, 105.11)	162.89% (39.92%, 333.53%)	3488.60 (2576.52, 4952.74)	180.89% (31.85%, 397.45%)
**Eastern Sub-Saharan Africa**	**83.36 (76.74, 93.07)**	− **28.90% (**− **45.36%, 2.36%)**	**3911.17 (3554.46, 4506.58)**	− **31.43% (**− **45.99%, **− **5.35%)**
Burundi	34.44 (27.52, 43.09)	− 75.21% (− 90.44%, − 47.85%)	1469.14 (1199.24, 1789.34)	− 78.36% (− 91.45%, − 54.79%)
Comoros	12.40 (8.17, 18.53)	− 13.88% (− 41.73%, 70.23%)	420.81 (273.47, 624.63)	− 15.97% (− 45.07%, 84.12%)
Djibouti	89.88 (67.24, 122.63)	573.68% (335.37%, 1071.98%)	4035.99 (2954.14, 5674.65)	795.31% (464.61%, 1470.36%)
Eritrea	42.90 (33.49, 53.56)	22.06% (− 27.07%, 94.07%)	1735.93 (1330.68, 2185.94)	15.41% (− 34.23%, 84.09%)
Ethiopia	38.73 (33.41, 45.83)	− 4.52% (− 38.88%, 44.83%)	1697.87 (1450.16, 2000.08)	− 10.16% (− 41.83%, 34.23%)
Kenya	123.01 (109.29, 139.35)	29.91% (− 18.46%, 113.99%)	5656.34 (5001.53, 6409.05)	17.22% (− 22.90%, 80.77%)
Madagascar	22.94 (17.54, 29.54)	64.80% (22.68%, 128.52%)	946.06 (725.18, 1231.84)	80.94% (32.04%, 155.17%)
Malawi	117.62 (102.97, 137.34)	− 32.41% (− 57.26%, 10.99%)	5669.32 (4942.01, 6691.23)	− 35.32% (− 57.87%, 2.09%)
Mozambique	278.92 (232.79, 357.85)	818.37% (577.94%, 1143.18%)	13,916.50 (11,468.42, 18,127.08)	903.32% (629.37%, 1223.18%)
Rwanda	43.49 (37.19, 51.03)	− 14.31% (− 57.35%, 39.67%)	1890.90 (1628.31, 2221.61)	− 18.68% (− 60.00%, 33.57%)
Somalia	38.61 (29.06, 50.63)	95.49% (36.29%, 189.71%)	1557.02 (1203.75, 1998.51)	121.40% (55.05%, 225.87%)
South Sudan	71.26 (35.83, 134.10)	354.58% (108.52%, 860.11%)	3344.80 (1605.88, 6592.53)	434.91% (123.05%, 1082.61%)
United Republic of Tanzania	71.64 (61.10, 84.34)	− 54.07% (− 68.30%, − 29.06%)	3344.67 (2864.06, 4009.68)	− 56.29% (− 69.21%, − 35.33%)
Uganda	81.73 (69.49, 99.48)	− 85.02% (− 88.13%, − 77.84%)	3978.66 (3310.24, 4955.23)	− 84.90% (− 87.78%, − 78.82%)
Zambia	162.74 (142.18, 189.39)	− 12.66% (− 42.98%, 41.99%)	7857.36 (6866.24, 9262.60)	− 17.09% (− 45.85%, 26.06%)
**Southern Sub-Saharan Africa**	**237.92 (212.64, 279.30)**	**542.00% (283.30%, 855.70%)**	**11,711.17 (10,265.58, 14,089.39)**	**538.35% (284.94%, 820.69%)**
Botswana	261.71 (222.49, 323.75)	147.74% (55.40%, 276.97%)	12,284.24 (10,323.94, 15,285.87)	123.51% (47.08%, 222.73%)
Lesotho	559.40 (480.85, 682.46)	1308.37% (869.35%, 1828.26%)	26,530.50 (22,530.83, 32,807.37)	1227.93% (832.65%, 1653.43%)
Namibia	180.09 (156.44, 212.96)	589.01% (375.53%, 916.19%)	8441.76 (7350.01, 9882.42)	548.20% (365.20%, 784.93%)
South Africa	240.15 (210.62, 289.65)	1406.98% (1091.33%, 1845.29%)	11,987.64 (10,295.82, 14,664.28)	1556.90% (1285.78%, 1918.88%)
Eswatini	340.59 (297.11, 402.25)	1947.93% (1338.16%, 3019.66%)	16,728.30 (14,591.41, 19,923.44)	2413.93% (1675.26%, 3517.61%)
Zimbabwe	181.34 (164.84, 202.64)	42.35% (− 37.72%, 172.44%)	8437.46 (7680.40, 9346.22)	30.45% (− 41.95%, 144.32%)
**Western Sub-Saharan Africa**	**52.17 (46.39, 59.27)**	**80.10% (34.20%, 129.21%)**	**2331.39 (2042.49, 2721.33)**	**76.87% (29.67%, 124.78%)**
Benin	30.98 (25.27, 37.84)	139.87% (80.89%, 207.73%)	1299.34 (1041.43, 1633.62)	185.26% (17.86%, 267.77%)
Burkina Faso	28.53 (23.76, 33.93)	− 82.63% (− 88.04%, − 73.01%)	1161.43 (972.29, 1399.04)	− 85.32% (− 89.78%, − 77.78%)
Cameroon	115.16 (102.99, 133.11)	376.73% (262.12%, 511.13%)	5239.84 (4643.06, 6158.28)	394.38% (276.12%, 533.88%)
Cabo Verde	16.61 (11.60, 27.68)	− 15.31% (− 51.59%, 54.91%)	643.05 (414.71, 1218.40)	− 18.83% (− 57.64%, 72.81%)
Chad	53.83 (41.02, 71.86)	110.57% (33.81%, 201.36%)	2387.61 (1767.55, 3269.09)	113.68% (31.85%, 214.04%)
Cote d'Ivoire	69.41 (58.89, 80.67)	− 22.48% (− 69.68%, 53.57%)	3089.00 (2623.77, 3656.46)	− 30.19% (− 72.52%, 39.66%)
The Gambia	71.19 (49.90, 98.27)	623.06% (376.69%, 960.76%)	3270.76 (2269.69, 4637.00)	736.91% (460.94%, 1116.21%)
Ghana	60.86 (52.52, 71.98)	108.42% (58.26%, 172.52%)	2740.17 (2315.54, 3335.45)	110.04% (59.85%, 174.93%)
Guinea	60.46 (46.91, 77.11)	134.21% (74.33%, 214.87%)	2665.44 (2014.63, 3532.42)	176.70% (103.17%, 276.43%)
Guinea-Bissau	83.79 (53.75, 129.15)	262.46% (124.14%, 472.46%)	3797.83 (2395.76, 5973.21)	302.17% (149.62%, 538.01%)
Liberia	49.99 (39.72, 62.67)	170.77% (76.53%, 294.27%)	2206.10 (1750.78, 2830.13)	192.31% (82.24%, 353.24%)
Mali	37.79 (30.15, 46.63)	93.08% (19.96%, 178.46%)	1675.86 (1302.48, 2103.45)	101.66% (16.53%, 197.63%)
Mauritania	9.78 (6.71, 15.05)	− 45.82% (− 73.43%, − 11.19%)	329.37 (222.28, 546.82)	− 49.23% (− 75.45%, − 11.99%)
Niger	22.29 (17.51, 27.56)	31.24% (− 4.49%, 79.59%)	863.32 (665.74, 1070.23)	30.06% (− 7.00%, 80.32%)
Nigeria	49.02 (40.15, 59.14)	233.21% (164.18%, 315.36%)	2213.38 (1784.24, 2733.04)	243.51% (172.48%, 324.13%)
Sao Tome and Principe	13.84 (9.59, 18.82)	− 7.95% (− 31.10%, 32.15%)	443.90 (319.45, 594.56)	− 12.26% (− 35.81%, 28.11%)
Senegal	23.56 (19.00, 28.63)	56.60% (19.70%, 103.53%)	950.80 (764.56, 1167.08)	61.78% (22.41%, 111.11%)
Sierra Leone	52.58 (42.97, 66.98)	292.84% (174.40%, 461.21%)	2389.44 (1914.80, 3093.87)	357.30% (203.86%, 581.38%)
Togo	61.50 (48.42, 77.04)	174.47% (79.42%, 286.38%)	2704.02 (2107.92, 3469.07)	187.25% (78.67%, 317.87%)
**Andean Latin America**	**12.70 (10.24, 16.85)**	**1.58% (**− **20.54%, 37.56%)**	**503.85 (401.14, 708.30)**	**14.54% (**− **14.30%, 61.33%)**
Bolivia	17.56 (10.62, 38.62)	− 17.28% (− 53.86%, 87.16%)	615.32 (324.23, 1677.97)	− 14.05% (− 59.28%, 129.57%)
Ecuador	13.34 (15.56, 11.64)	37.89% (20.45%, 61.69%)	545.38 (488.23, 621.86)	66.98% (48.36%, 92.97%)
Peru	11.10 (8.30, 14.31)	− 2.81% (− 27.48%, 31.82%)	451.98 (337.91, 603.38)	8.89% (− 19.75%, 51.86%)
**Tropical Latin America**	**9.50 (8.88, 10.46)**	− **16.20% (**− **20.73%, **− **11.50%)**	**406.91 (375.23, 443.82)**	− **8.64% (**− **13.50%, **− **3.44%)**
Brazil	9.39 (8.78, 10.39)	− 17.30% (− 21.86%, − 12.51%)	403.64 (372.54, 440.21)	− 9.79% (− 14.52%, − 4.58%)
Paraguay	13.60 (10.18, 17.38)	32.09% (− 0.83%, 72.96%)	532.45 (404.17, 670.43)	46.96% (9.72%, 92.70%)
**Central Latin America**	**10.04 (9.04, 11.34)**	− **23.76% (**− **31.97%, **− **12.26%)**	**409.15 (373.42, 454.98)**	− **10.78% (**− **18.60%, **− **1.08%)**
Colombia	9.04 (7.91, 10.37)	− 9.67% (− 21.85%, 4.56%)	384.76 (342.37, 433.00)	8.43% (− 4.01%, 22.65%)
Costa Rica	6.50 (5.56, 7.79)	− 32.61% (− 43.04%, − 15.58%)	265.23 (231.41, 310.18)	− 22.49% (− 32.27%, − 7.22%)
El Salvador	18.32 (13.26, 23.37)	67.42% (18.91%, 119.76%)	730.34 (531.30, 918.02)	82.99% (30.74%, 137.41%)
Guatemala	14.09 (10.88, 17.13)	18.40% (− 34.31%, 48.08%)	520.55 (427.45, 617.15)	13.04% (− 29.68%, 39.20%)
Honduras	6.94 (4.81, 9.88)	− 13.14% (− 35.80%, 17.47%)	258.08 (185.09, 362.04)	− 22.07% (− 41.18%, 4.02%)
Mexico	8.72 (7.71, 10.40)	− 41.87% (− 49.66%, − 17.41%)	349.75 (314.77, 404.93)	− 30.49% (− 37.67%, − 10.60%)
Nicaragua	17.02 (13.13, 21.63)	41.03% (6.68%, 88.13%)	657.71 (494.23, 831.97)	56.89% (17.83%, 107.48%)
Panama	17.03 (15.62, 18.64)	1.29% (− 8.99%, 16.47%)	806.03 (744.65, 871.28)	17.36% (7.13%, 33.09%)
Venezuela	13.70 (11.55, 16.30)	1.82% (− 15.67%, 23.26%)	568.84 (489.92, 666.26)	15.72% (− 1.87%, 37.28%)
**Southern Latin America**	**8.28 (7.50, 8.78)**	**2.71% (**− **8.13%, 10.04%)**	**349.46 (318.69, 385.34)**	**15.79% (5.65%, 27.09%)**
Argentina	9.22 (8.00, 9.85)	24.34% (0.00%, 35.47%)	394.38 (347.38, 438.26)	34.49% (14.88%, 49.59%)
Chile	6.22 (5.80, 6.78)	− 41.56% (− 46.12%, − 28.72%)	252.53 (231.10, 287.06)	− 28.40% (− 34.45%, − 15.28%)
Uruguay	7.93 (7.24, 8.57)	12.75% (1.23%, 23.39%)	314.10 (287.15, 343.50)	24.67% (13.30%, 35.32%)
**Caribbean**	**20.97 (18.01, 24.39)**	**14.41% (**− **8.90%, 37.02%)**	**937.98 (797.21, 1114.91)**	**16.72% (**− **7.76%, 42.45%)**
Antigua and Barbuda	11.32 (10.17, 12.50)	− 32.48% (− 39.09%, − 24.66%)	447.64 (404.58, 490.21)	− 34.03% (− 39.26%, − 27.75%)
The Bahamas	26.59 (24.44, 28.54)	− 6.75% (− 12.33%, 0.05%)	1203.30 (1093.39, 1307.07)	− 6.50% (− 11.48%, − 1.14%)
Barbados	12.71 (11.23, 14.36)	− 40.86% (− 47.24%, − 33.60%)	503.67 (449.49, 562.57)	− 40.96% (− 46.39%, − 34.65%)
Belize	24.29 (22.38, 26.22)	− 2.66% (− 9.66%, 5.13%)	1081.54 (991.21, 1173.07)	7.33% (0.51%, 14.45%)
Bermuda	7.70 (7.17, 8.31)	− 63.96% (− 65.93%, − 61.35%)	344.31 (316.48, 372.73)	− 61.61% (− 63.51%, − 59.29%)
Cuba	6.06 (5.15, 7.04)	2.18% (− 12.64%, 19.85%)	251.55 (217.56, 292.28)	15.31% (0.74%, 32.51%)
Dominica	14.12 (12.09, 16.48)	− 29.65% (− 39.36%, − 17.26%)	549.13 (478.58, 627.98)	− 27.31% (− 36.43%, − 16.75%)
Dominican Republic	17.25 (13.69, 22.52)	69.30% (15.75%, 130.69%)	703.90 (545.60, 951.56)	68.32% (11.12%, 135.21%)
Grenada	12.77 (11.20, 14.12)	− 37.72% (− 45.37%, − 29.84%)	467.71 (413.68, 517.51)	− 40.15% (− 47.10%, − 32.78%)
Guyana	29.29 (25.99, 32.94)	16.11% (1.19%, 33.08%)	1312.11 (1168.71, 1469.14)	37.24% (21.42%, 55.27%)
Haiti	56.71 (45.81, 69.04)	4.06% (− 30.49%, 49.43%)	2409.72 (1934.29, 2994.41)	− 0.53% (− 35.29%, 47.03%)
Jamaica	19.12 (16.80, 21.69)	15.75% (3.30%, 30.80%)	798.91 (708.16, 892.18)	24.17% (13.06%, 37.79%)
Puerto Rico	6.44 (5.84, 7.15)	− 71.99% (− 74.54%, − 68.65%)	285.88 (257.12, 316.30)	− 75.03% (− 77.22%, − 72.42%)
Saint Lucia	10.00 (8.69, 11.58)	− 47.49% (− 54.34%, − 39.44%)	379.60 (333.98, 433.38)	− 45.62% (− 52.08%, − 37.70%)
Saint Vincent and the Grenadines	22.77 (20.83, 24.77)	− 22.65% (− 29.14%, − 15.61%)	950.42 (866.00, 1032.73)	− 15.36% (− 21.54%, − 8.06%)
Suriname	21.56 (19.30, 24.08)	− 9.24% (− 17.71%, 0.48%)	930.69 (841.42, 1022.52)	− 2.59% (− 10.16%, 6.00%)
Trinidad and Tobago	15.81 (14.03, 17.95)	− 4.54% (− 14.90%, 8.33%)	692.75 (620.16, 775.76)	10.22% (0.47%, 21.96%)
United States Virgin Islands	9.62 (8.53, 10.87)	− 21.09% (− 30.42%, − 10.01%)	383.58 (343.31, 425.52)	− 18.55% (− 26.92%, − 8.82%)
**Central Europe**	**3.83 (3.26, 4.40)**	− **33.90% (**− **43.45%, **− **24.36%)**	**131.58 (112.22, 150.40)**	− **32.55% (**− **41.47%, **− **23.22%)**
Albania	1.70 (1.22, 2.33)	− 16.64% (− 41.34%, 14.83%)	62.12 (45.61, 82.82)	− 16.19% (− 37.25%, 11.47%)
Bosnia and Herzegovina	3.06 (2.15, 3.94)	− 15.13% (− 37.44%, 11.18%)	103.95 (74.08, 133.18)	− 12.14% (− 35.45%, 14.37%)
Bulgaria	4.63 (3.33, 5.96)	11.88% (− 20.86%, 43.72%)	173.39 (122.55, 221.38)	11.43% (− 18.65%, 42.09%)
Croatia	2.16 (1.66, 2.74)	− 48.71% (− 61.04%, − 32.69%)	72.57 (57.18, 92.12)	− 44.38% (− 56.59%, − 28.13%)
Czech Republic	2.48 (2.03, 3.03)	− 49.54% (− 58.85%, − 38.22%)	83.01 (68.18, 100.26)	− 47.43% (− 56.58%, − 36.27%)
Hungary	3.20 (2.61, 3.94)	− 48.62% (− 57.95%, − 37.17%)	111.83 (91.65, 137.10)	− 49.30% (− 58.08%, − 38.26%)
Macedonia	3.20 (2.38, 4.22)	− 10.65% (− 33.38%, 18.35%)	106.75 (79.14, 137.11)	− 15.48% (− 35.70%, 11.75%)
Montenegro	2.72 (2.22, 3.44)	− 5.56% (− 31.93%, 17.95%)	99.22 (81.46, 120.29)	− 5.55% (− 27.83%, 16.54%)
Poland	3.48 (2.74, 4.36)	− 47.20% (− 58.00%, − 33.67%)	113.95 (91.83, 141.72)	− 45.67% (− 55.91%, − 32.50%)
Romania	6.26 (4.56, 7.76)	− 12.81% (− 37.86%, 8.31%)	223.93 (163.04, 277.39)	− 13.62% (− 33.38%, 6.18%)
Serbia	4.84 (3.64, 6.27)	− 23.67% (− 42.42%, 1.26%)	165.30 (122.80, 213.54)	− 21.98% (− 40.28%, 4.23%)
Slovakia	2.90 (1.93, 3.75)	− 25.71% (− 47.34%, − 1.23%)	100.32 (69.52, 128.77)	− 25.72% (− 46.22%, − 1.51%)
Slovenia	1.63 (1.21, 2.17)	− 55.08% (− 69.03%, − 35.33%)	56.30 (43.51, 73.89)	− 52.07% (− 66.49%, − 32.87%)
**Eastern Europe**	**8.25 (6.92, 9.43)**	**44.58% (24.34%, 64.22%)**	**402.54 (330.91, 467.77)**	**106.62% (78.68%, 131.89%)**
Belarus	5.23 (4.37, 6.48)	− 8.44% (− 24.04%, 13.72%)	216.04 (185.86, 259.82)	12.88% (− 4.31%, 34.25%)
Estonia	4.37 (3.65, 5.23)	− 30.83% (− 42.69%, − 16.68%)	188.38 (162.39, 219.80)	− 4.86% (− 18.72%, 13.58%)
Latvia	5.60 (4.91, 6.56)	1.97% (− 12.29%, 22.63%)	236.84 (212.28, 273.25)	35.68% (20.51%, 57.84%)
Lithuania	3.86 (3.16, 4.62)	− 34.59% (− 45.99%, − 21.10%)	134.42 (111.81, 160.52)	− 29.73% (− 41.08%, − 15.91%)
Moldova	6.42 (5.73, 7.26)	− 2.54% (− 13.40%, 11.09%)	285.28 (256.60, 318.19)	25.81% (13.85%, 41.26%)
Russian Federation	8.28 (6.76, 9.68)	67.29% (39.98%, 90.71%)	414.40 (331.98, 488.36)	142.46% (102.24%, 174.29%)
Ukraine	9.33 (7.90, 10.96)	22.88% (3.91%, 89.89%)	438.55 (365.00, 514.50)	69.36% (45.92%, 141.45%)
**North Africa and Middle East**	**2.44 (1.91, 3.26)**	**4.60% (**− **15.77%, 47.22%)**	**97.39 (74.92, 139.08)**	**17.28% (**− **8.08%, 66.20%)**
Afghanistan	4.43 (2.05, 6.94)	− 6.36% (− 34.91%, 44.85%)	156.78 (66.43, 261.34)	− 10.03% (− 39.33%, 49.88%)
Algeria	2.53 (1.68, 4.76)	− 34.69% (− 55.58%, 17.21%)	92.71 (60.86, 195.95)	− 32.63% (− 54.06%, 27.19%)
Bahrain	1.41 (1.12, 1.84)	− 44.74% (− 57.34%, − 23.95%)	44.50 (36.14, 55.74)	− 43.91% (− 55.18%, − 28.08%)
Egypt	0.82 (0.57, 1.17)	− 22.76% (− 45.70%, 6.27%)	35.26 (26.32, 46.98)	− 18.99% (− 34.97%, 0.55%)
Iran	1.46 (1.14, 1.76)	− 12.33% (− 33.28%, 13.78%)	56.21 (45.65, 69.45)	− 2.04% (− 18.39%, 23.51%)
Iraq	1.35 (0.98, 1.79)	− 13.20% (− 39.63%, 27.63%)	53.41 (39.23, 70.81)	− 10.90% (− 35.17%, 25.87%)
Jordan	1.13 (0.86, 1.45)	− 38.87% (− 53.86%, − 10.54%)	42.69 (33.59, 53.71)	− 32.74% (− 47.20%, − 8.96%)
Kuwait	0.75 (0.56, 1.01)	− 42.16% (− 56.96%, − 17.81%)	29.53 (22.87, 39.78)	− 35.19% (− 48.27%, − 16.12%)
Lebanon	1.86 (1.06, 3.86)	− 31.68% (− 59.76%, 35.01%)	74.38 (40.32, 171.39)	− 29.27% (− 60.05%, 44.27%)
Libya	2.62 (1.55, 5.35)	9.33% (− 33.88%, 134.34%)	101.81 (55.50, 235.94)	19.57% (− 30.51%, 182.69%)
Morocco	4.63 (2.79, 8.11)	− 4.74% (− 37.71%, 70.83%)	166.10 (94.40, 330.24)	− 1.19% (− 37.98%, 90.89%)
Palestine	1.71 (1.08, 2.06)	− 22.10% (− 43.55%, 10.37%)	56.52 (42.47, 68.45)	− 20.02% (− 38.46%, 7.17%)
Oman	3.15 (2.33, 4.22)	37.01% (− 11.10%, 115.05%)	116.23 (83.65, 158.83)	58.59% (3.81%, 140.56%)
Qatar	1.27 (0.98, 1.65)	− 49.45% (− 62.30%, − 30.36%)	36.60 (29.15, 46.83)	− 47.39% (− 58.35%, − 32.17%)
Saudi Arabia	1.90 (1.25, 3.52)	30.72% (− 27.53%, 150.70%)	80.10 (51.53, 152.26)	48.84% (− 13.85%, 188.35%)
Sudan	11.90 (7.05, 21.04)	309.78% (97.05%, 807.76%)	561.37 (317.43, 994.85)	376.13% (110.97%, 947.30%)
Syria	0.97 (0.74, 1.29)	− 22.20% (− 43.77%, 17.06%)	40.02 (30.94, 52.51)	− 20.23% (− 38.68%, 9.77%)
Tunisia	2.08 (1.12, 5.13)	13.04% (− 39.34%, 160.00%)	87.33 (45.55, 231.53)	24.94% (− 34.18%, 208.54%)
Turkey	1.58 (1.14, 1.94)	− 32.55% (− 48.99%, 4.31%)	50.71 (40.31, 61.15)	− 33.65% (− 47.99%, − 3.67%)
United Arab Emirates	3.72 (1.34, 14.32)	− 23.05% (− 68.34%, 215.48%)	100.45 (42.45, 333.39)	− 14.80% (− 62.03%, 187.14%)
Yemen	3.07 (1.43, 7.66)	− 1.09% (− 53.59%, 165.07%)	121.14 (54.82, 337.34)	7.48% (− 56.34%, 209.47%)
**Central Asia**	**4.70 (4.19, 5.33)**	− **21.65% (**− **29.35%, **− **10.90%)**	**172.71 (153.01, 197.65)**	− **15.40% (**− **23.14%, **− **5.57%)**
Armenia	4.37 (3.58, 5.21)	− 22.92% (− 37.98%, − 6.26%)	153.89 (128.19, 184.77)	− 21.45% (− 35.09%, − 5.30%)
Azerbaijan	3.48 (2.67, 4.98)	− 24.35% (− 42.49%, − 0.92%)	121.15 (94.08, 162.20)	− 25.94% (− 42.56%, − 5.10%)
Georgia	4.55 (3.66, 5.47)	− 25.96% (− 41.00%, − 8.68%)	169.72 (134.90, 203.37)	− 20.93% (− 36.35%, − 2.82%)
Kazakhstan	4.95 (4.25, 5.86)	− 31.18% (− 41.18%, − 15.12%)	188.40 (163.06, 221.13)	− 17.70% (− 28.29%, − 4.84%)
Kyrgyzstan	5.75 (4.79, 6.65)	− 24.10% (− 35.00%, − 11.58%)	212.30 (181.60, 243.80)	− 16.33% (− 27.31%, − 3.90%)
Mongolia	7.17 (5.19, 9.63)	− 29.10% (− 47.96%, − 1.41%)	220.25 (163.72, 301.87)	− 30.90% (− 49.33%, − 3.30%)
Tajikistan	3.04 (2.41, 4.19)	− 34.45% (− 50.12%, 5.37%)	110.17 (89.41, 151.77)	− 32.46% (− 46.94%, 0.46%)
Turkmenistan	4.59 (3.49, 5.78)	− 17.12% (− 34.67%, 3.89%)	183.66 (142.73, 229.10)	− 11.37% (− 29.01%, 9.47%)
Uzbekistan	5.15 (4.20, 6.14)	4.35% (− 15.17%, 25.54%)	187.40 (153.69, 223.25)	5.64% (− 13.14%, 25.09%)
**South Asia**	**5.63 (4.75, 7.10)**	**1.35% (**− **17.53%, 29.91%)**	**226.23 (194.48, 276.96)**	**17.54% (**− **3.87%, 51.73%)**
Bangladesh	3.24 (1.96, 4.76)	− 55.45% (− 69.36%, − 23.16%)	111.32 (70.83, 162.14)	− 52.79% (− 68.31%, − 15.42%)
Bhutan	6.76 (2.73, 23.76)	− 15.31% (− 63.81%, 188.18%)	272.33 (94.54, 1058, 62)	− 2.60% (− 65.27%, 270.50%)
India	6.13 (5.16, 7.53)	8.29% (− 12.18%, 39.20%)	249.84 (215.57, 296.76)	26.57% (4.36%, 61.36%)
Nepal	8.09 (3.33, 25.94)	4.07% (− 52.80%, 264.87%)	324.41 (123.59, 1072.81)	16.98% (− 52.79%, 341.22%)
Pakistan	3.15 (1.93, 6.82)	23.08% (− 25.89%, 157.60%)	123.17 (71.90, 284.29)	30.40% (− 24.92%, 193.26%)
**Southeast Asia**	**8.14 (7.10, 9.92)**	**29.60% (7.10%, 66.54%)**	**351.79 (308.94, 412.06)**	**60.39% (30.13%, 107.11%)**
Cambodia	11.91 (9.10, 15.64)	39.77% (− 0.13%, 135.66%)	501.00 (378.94, 661.17)	68.48% (18.25%, 185.47%)
Indonesia	5.09 (3.82, 7.69)	− 2.35% (− 21.93%, 30.31%)	220.12 (177.70, 285.30)	21.58% (− 2.80%, 65.15%)
Laos	9.65 (3.85, 42.00)	6.57% (− 59.04%, 540.01%)	404.99 (155.43, 1890.97)	27.94% (− 54.89%, 695.40%)
Malaysia	6.30 (4.82, 7.76)	− 7.68% (− 27.59%, 21.11%)	227.39 (181.49, 278.72)	9.64% (− 13.26%, 43.71%)
Maldives	2.04 (1.63, 2.55)	− 64.46% (− 75.85%, − 15.26%)	66.88 (54.19, 83.99)	− 66.90% (− 77.22%, − 26.26%)
Mauritius	7.60 (6.69, 8.59)	20.84% (5.05%, 40.15%)	327.80 (288.82, 364.92)	62.93% (43.67%, 86.12%)
Myanmar	10.37 (8.37, 13.80)	28.21% (− 9.15%, 105.08%)	477.48 (380.26, 602.12)	63.54% (12.10%, 170.96%)
Philippines	7.29 (6.34, 8.65)	26.52% (3.87%, 52.75%)	343.06 (303.12, 392.93)	56.85% (33.29%, 86.36%)
Sri Lanka	2.21 (1.54, 2.99)	− 6.91% (− 38.32%, 33.70%)	77.68 (57.53, 100.70)	− 11.79% (− 35.47%, 19.00%)
Seychelles	10.60 (8.87, 13.18)	− 15.40% (− 30.88%, 3.11%)	348.87 (292.97, 436.47)	− 10.87% (− 26.94%, 9.33%)
Thailand	19.16 (15.36, 26.49)	115.99% (64.87%, 211.28%)	874.41 (700.62, 1188.89)	172.30% (109.28%, 277.54%)
Timor-Leste	19.33 (4.40, 95.24)	174.44% (− 35.40%, 1109.11%)	834.18 (155.16, 4211.48)	225.50% (− 34.16%, 1240.19%)
Vietnam	7.50 (5.96, 9.13)	19.84% (− 12.03%, 67.47%)	296.64 (244.96, 360.91)	45.83% (8.99%, 101.52%)
**East Asia**	**3.84 (2.73, 4.63)**	**14.06% (**− **40.31%, 58.39%)**	**142.99 (107.56, 169.76)**	**28.48% (**− **29.65%, 76.42%)**
China	3.81 (2.69, 4.62)	16.76% (− 40.57%, 63.83%)	142.25 (105.82, 169.51)	31.46% (− 29.47%, 83.03%)
North Korea	6.05 (3.56, 13.16)	6.18% (− 37.96%, 149.25%)	229.09 (126.05, 573.21)	19.48% (− 37.28%, 207.55%)
Taiwan (Province of China)	3.03 (2.42, 3.98)	− 51.41% (− 61.70%, − 35.89%)	97.86 (78.99, 127.36)	− 49.23% (− 58.88%, − 32.26%)
**Oceania**	**38.57 (18.31, 92.09)**	**314.64% (91.02%, 878.79%)**	**1724.76 (735.51, 4282.37)**	**443.59% (120.71%, 1259.71%)**
American Samoa	5.81 (4.38, 7.67)	13.31% (− 20.81%, 57.86%)	202.66 (148.88, 277.02)	23.47% (− 14.54%, 77.85%)
Federated States of Micronesia	53.28 (10.88, 229.90)	284.47% (− 15.23%, 1543.90%)	2264.81 (365.90, 10,140.15)	384.02% (− 10.78%, 1859.98%)
Fiji	12.71 (5.91, 16.78)	− 10.90% (− 38.13%, 22.91%)	408.17 (207.54, 538.39)	− 11.31% (− 36.53%, 24.62%)
Guam	6.32 (4.31, 9.91)	24.85% (− 20.53%, 104.13%)	268.31 (174.50, 438.74)	65.04% (− 1.06%, 178.95%)
Kiribati	41.03 (30.42, 52.08)	− 12.59% (− 33.84%, 13.58%)	1252.79 (919.07, 1603.69)	− 17.05% (− 38.93%, 9.41%)
Marshall Islands	16.54 (7.02, 58.44)	32.45% (− 36.41%, 326.74%)	647.82 (249.86, 2661.69)	57.28% (− 30.85%, 445.42%)
Northern Mariana Islands	8.95 (6.87, 11.38)	− 5.37% (− 32.08%, 32.67%)	296.95 (219.43, 388.90)	0.67% (− 30.54%, 45.49%)
Papua New Guinea	45.74 (20.00, 116.40)	542.21% (148.23%, 1561.00%)	2070.62 (792.98, 5451.29)	729.97% (184.01%, 2165.94%)
Samoa	12.56 (4.76, 55.75)	56.91% (− 36.27%, 479.52%)	517.08 (174.15, 2517.53)	81.36% (− 32.76%, 617.33%)
Solomon Islands	20.71 (9.82, 66.02)	32.46% (− 29.70%, 268.75%)	816.87 (326.94, 2801.87)	39.85% (− 31.04%, 315.47%)
Tonga	11.15 (8.08, 15.48)	− 6.17% (− 33.62%, 34.50%)	362.53 (256.17, 519.11)	− 3.44% (− 34.71%, 43.38%)
Vanuatu	14.02 (6.13, 53.93)	60.82% (− 21.53%, 382.79%)	540.88 (222.66, 2279.47)	79.09% (− 18.41%, 474.56%)
**High-income Asia Pacific**	**1.57 (1.31, 1.70)**	− **35.59% (**− **48.49, **− **29.97%)**	**60.93 (52.14, 70.49)**	− **25.04% (**− **39.08%, **− **18.69%)**
Brunei	6.71 (5.70, 8.11)	− 29.03% (− 42.22%, − 10.20%)	211.07 (177.49, 256.39)	− 26.83% (− 41.39%, − 5.77%)
Japan	1.56 (1.25, 1.71)	− 26.53% (− 44.19%, − 20.90%)	61.04 (49.19, 70.60)	− 10.37% (− 32.04%, − 3.24%)
Singapore	1.77 (1.58, 2.00)	− 60.57% (− 64.57%, − 54.98%)	66.86 (58.75, 79.32)	− 53.29% (− 58.61%, − 45.70%)
South Korea	1.69 (1.37, 2.04)	− 52.44% (− 65.04%, − 42.09%)	61.38 (51.45, 74.89)	− 51.31% (− 59.84%, − 41.46%)
**High-income North America**	**3.01 (2.76, 3.11)**	− **67.30% (**− **69.29%, **− **64.76%)**	**147.70 (129.09, 172.00)**	− **67.29% (**− **70.74%, **− **62.64%)**
Canada	1.77 (1.55, 1.92)	− 51.58% (− 56.49%, − 47.20%)	82.93 (71.22, 98.62)	− 49.21% (− 54.76%, − 42.80%)
Greenland	6.00 (4.97, 7.34)	− 47.69% (− 58.46%, − 33.65%)	235.07 (195.80, 282.56)	− 43.39% (− 55.62%, − 28.74%)
United States	3.16 (2.91, 3.27)	− 67.90% (− 69.92%, − 65.38%)	155.17 (135.60, 180.95)	− 67.92% (− 71.42%, − 63.25%)
**Western Europe**	**1.96 (1.82, 2.07)**	− **56.77% (**− **58.91%, **− **54.12%)**	**77.89 (70.56, 87.36)**	− **58.52% (**− **61.61%, **− **54.53%)**
Andorra	4.41 (2.03, 12.34)	− 1.64% (− 52.52%, 146.66%)	169.90 (67.54, 504.10)	0.21% (− 57.47%, 183.34%)
Austria	1.71 (1.55, 2.08)	− 61.65% (− 65.32%, − 49.42%)	66.65 (58.16, 80.93)	− 53.74% (− 59.43%, − 41.03%)
Belgium	1.82 (1.62, 1.99)	− 49.20% (− 53.46%, − 44.48%)	71.84 (62.82, 82.41)	− 40.70% (− 46.98%, − 33.46%)
Cyprus	1.85 (1.33, 2.13)	− 20.97% (− 49.95%, 9.51%)	54.78 (43.87, 63.06)	− 16.71% (− 43.19%, 8.65%)
Denmark	2.06 (1.86, 2.69)	− 57.03% (− 61.52%, − 35.52%)	73.06 (64.72, 90.15)	− 50.83% (− 56.62%, − 31.11%)
Finland	1.05 (0.79, 1.17)	− 39.56% (− 52.90%, − 30.96%)	33.54 (28.56, 39.22)	− 35.22% (− 46.31%, − 27.22%)
France	2.05 (1.85, 2.24)	− 71.59% (− 73.98%, − 69.10%)	80.12 (71.13, 90.36)	− 74.72% (− 77.06%, − 71.96%)
Germany	1.88 (1.72, 2.04)	− 57.43% (− 60.73%, − 53.87%)	68.87 (61.78, 78.40)	− 58.87% (− 62.33%, − 54.29%)
Greece	1.69 (1.54, 1.90)	− 37.22% (− 42.38%, − 29.25%)	56.76 (51.15, 65.00)	− 32.85% (− 38.51%, − 23.75%)
Iceland	1.10 (0.93, 1.27)	− 56.02% (− 62.13%, − 46.19%)	38.70 (32.77, 45.94)	− 54.35% (− 60.63%, − 45.20%)
Ireland	1.63 (1.32, 1.86)	− 36.11% (− 50.78%, − 26.09%)	60.95 (47.21, 70.74)	− 32.69% (− 45.51%, − 22.13%)
Israel	1.65 (1.36, 1.82)	− 25.36% (− 46.41%, − 16.24%)	63.14 (53.10, 73.05)	− 18.46% (− 37.09%, − 6.92%)
Italy	1.89 (1.42, 2.02)	− 47.41% (− 62.61%, − 39.98%)	78.10 (63.46, 89.35)	− 56.58% (− 65.86%, − 48.98%)
Luxembourg	1.30 (1.14, 1.57)	− 62.18% (− 67.15%, − 54.85%)	49.72 (43.08, 59.33)	− 59.01% (− 64.22%, − 51.43%)
Malta	1.18 (1.02, 1.38)	− 50.82% (− 57.44%, − 42.08%)	47.05 (40.62, 55.58)	− 42.63% (− 50.19%, − 33.35%)
Netherlands	1.43 (1.27, 1.56)	− 59.47% (− 62.74%, − 56.02%)	55.25 (48.19, 63.24)	− 60.94% (− 64.92%, − 56.59%)
Norway	1.72 (1.55, 1.89)	− 48.27% (− 51.68%, − 44.00%)	62.34 (55.64, 72.07)	− 47.07% (− 50.94%, − 41.55%)
Portugal	4.85 (4.57, 5.15)	− 3.83% (− 10.72%, 4.69%)	216.36 (201.04, 233.76)	12.43% (3.60%, 24.13%)
Spain	2.23 (1.83, 2.38)	− 62.89% (− 68.05%, − 58.62%)	92.73 (80.63, 102.58)	− 68.77% (− 72.49%, − 64.19%)
Sweden	1.57 (1.42, 1.73)	− 41.73% (− 47.44%, − 37.02%)	53.20 (47.38, 61.45)	− 41.35% (− 47.54%, − 35.65%)
Switzerland	1.47 (1.30, 1.62)	− 40.99% (− 47.20%, − 25.20%)	61.24 (52.82, 73.30)	− 25.11% (− 33.81%, − 6.98%)
United Kingdom	1.83 (1.72, 2.27)	− 52.90% (− 55.03%, − 41.16%)	77.84 (68.85, 93.38)	− 43.97% (− 49.41%, − 31.63%)
**Australasia**	**1.38 (1.22, 1.50)**	− **64.41% (**− **67.31%, **− **61.43%)**	**57.37 (49.74, 66.45)**	− **65.36% (**− **68.58%, **− **61.29%)**
Australia	1.37 (1.19, 1.50)	− 63.84% (− 67.46%, − 60.73%)	57.02 (49.13, 66.25)	− 65.37% (− 68.81%, − 61.49%)
New Zealand	1.43 (1.27, 1.59)	− 66.87% (− 70.05%, − 58.59%)	59.62 (51.72, 69.79)	− 65.12% (− 69.06%, − 55.55%)

Unexpectedly, the Low-SDI quintile region had nearly three times more than the global level for both the ASMR and ASDR, with the incidence of the ASMR and ASDR being 34.33 (95% UI: 31.13–38.55)/100,000 people and 1579.77 (95% UI: 1410.49–1829.90)/100,000 people, respectively, in the Low-SDI quintile region and 2.24 (95% UI: 2.06–2.36)/100,000 people and 2207.76 (95% UI: 1850.96–2672.43)/100,000 people, respectively, in the High-SDI quintile region. In addition, both the ASMR and ASDR showed significant growth from 1990 to 2019 in the middle-SDI quintile, with an increase of 116.28% (95% UI: 75.02–146.32%) in the ASMR and 183.31% (95% UI: 134.83–222.39%) in the ASDR.

### Burden attributable to unsafe sex in various countries

**Figure 2:**
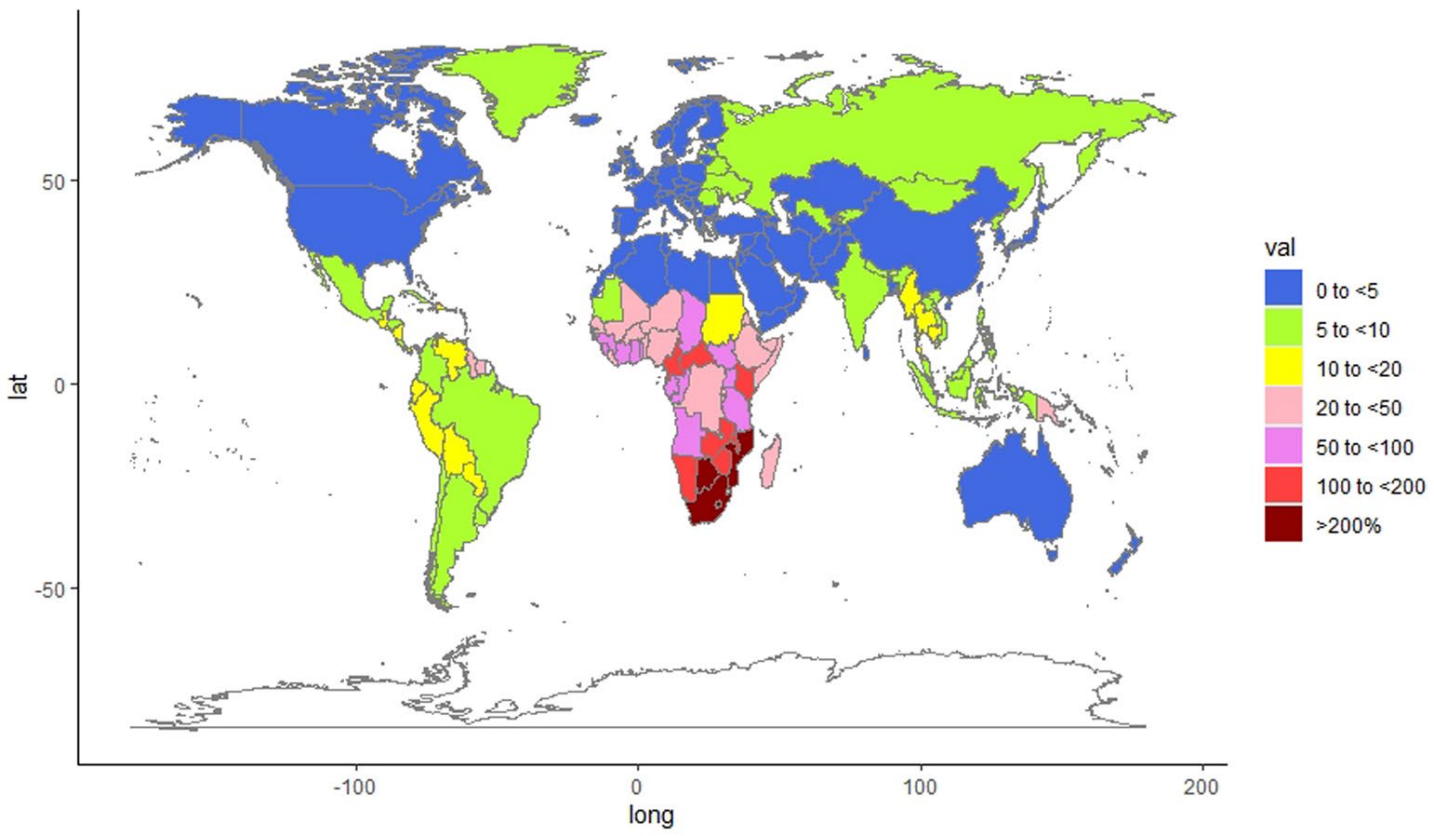
Global ASMR per 100,000 people attributable to unsafe sex in 2019.

**Figure 3:**
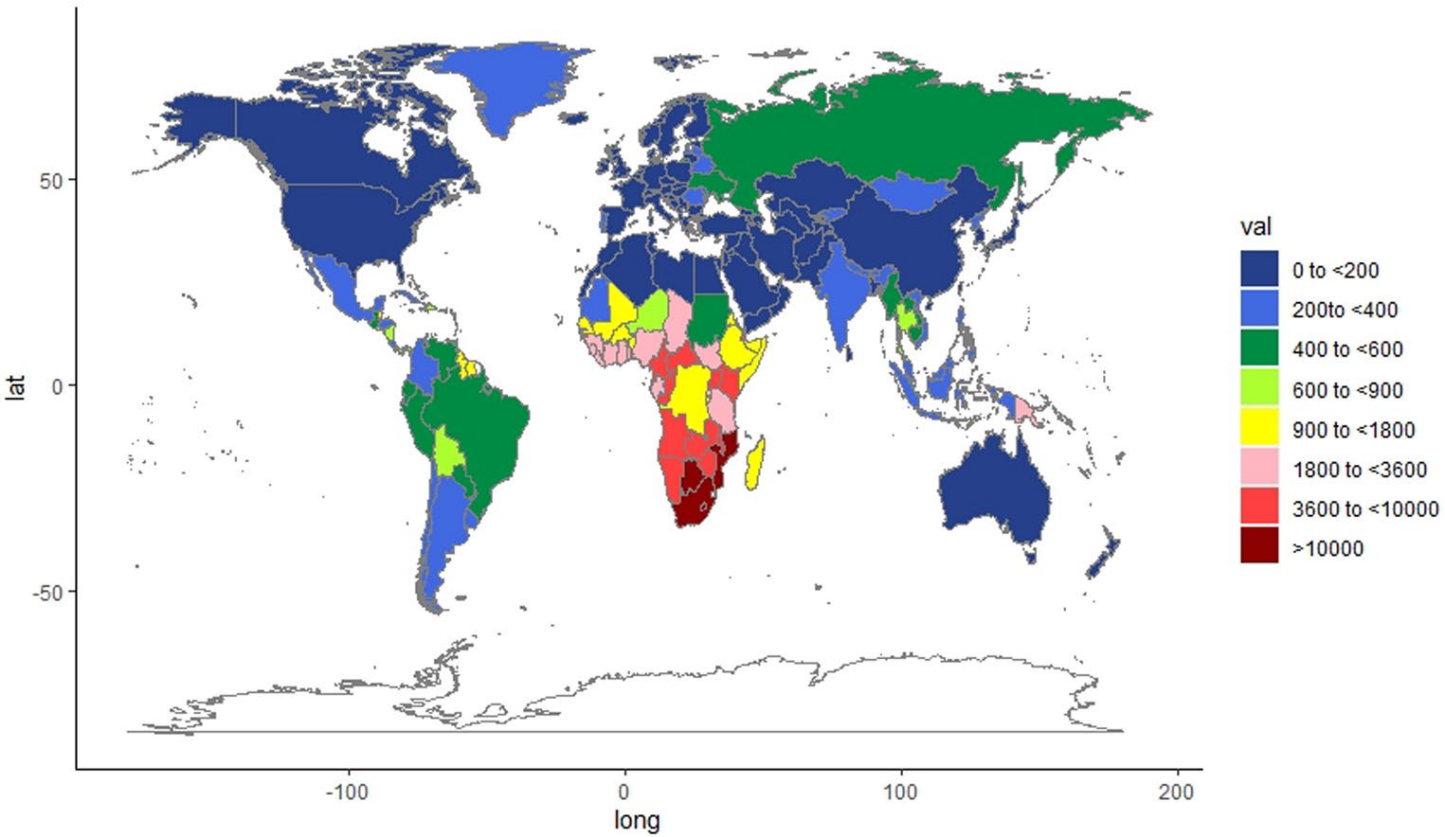
Global ASDR per 100,000 people attributable to unsafe sex in 2019.

As shown in Figs. 2, 3 and Table 1, among the 204 countries in the world, the three countries in southern sub-Saharan Africa, Lesotho [ASMR: 559.40 (95% UI: 480.85–682.46)/100,000 people and ASDR: 26,530.50 (95% UI: 22,530.83–32,807.37)/100,000 people], Eswatini [ASMR: 340.59 (95% UI: 297.11–420.25)/100,000 people and ASDR: 16,728.30 (95% UI: 14,591.41–19,923.44)/100,000 people] and South Africa [ASMR: 240.15 (95% UI: 210.62–289.65)/100,000 people and ASDR: 11,987.64 (95% UI: 10,295.82–14,664.28)/100,000 people] were the top three countries in the ASMRs and ASDRs in 2019 and the fastest growing countries of the ASMRs and ASDRs attributable to unsafe sex from 1990 to 2019. Kuwait [ASMR: 0.75 (95% UI: 0.56–1.01)/100,000 people and ASDR: 29.53 (95% UI: 22.87–39.78)/100,000 people], Egypt [ASMR: 0.82 (95% UI: 0.57–1.17)/100,000 people and ASDR: 35.26 (95% UI: 26.32–46.98)/100,000 people] and Syria [ASMR: 0.97 (95% UI: 0.74–1.29)/100,000 people and ASDR: 40.02 (95% UI: 30.94–52.51)/100,000 people] were the bottom three countries in the ASMRs and ASDRs attributable to unsafe sex in 2019. The percentage change in ASMRs and ASDRs attributable to unsafe sex decreased most in the countries of Uganda [ASMR: − 85.02% (95% UI: − 88.13%, − 77.84% and ASDR: − 84.90% (95% UI: − 87.78%, − 78.82%)] and Burkina Faso [ASMR: − 82.63% (95% UI: − 88.04%, − 73.01%) and ASDR: − 85.32% (95% UI: − 89.78%, − 77.78%)] from 1990 to 2019. Among the top 50 most populous countries released by the United Nations, Mozambique, South Africa and Kenya had the highest ASDR and ASMR attributed to unsafe sex in 2019. Meanwhile, Egypt, Iran and Iraq have the lowest ASDR and ASMR.

### Burden attributable to unsafe sex in sex and age

From 1990 to 2019, the trend of deaths and DALYs burden of both sex attributed to unsafe sex was also was consistent with the overall global situation (Fig. 1). In 2004, the deaths and DALYs attributed to unsafe sex in man were 610,000 (95% UI: 460,000–760,000) and 31 million (95% UI: 24–39), respectively, while the deaths and DALYs attributed to unsafe sex in woman were 1 million (95% UI: 0.8–1.3) and 52 million (95% UI: 41–65), respectively. The burdens of deaths and DALYs were generally higher in females than in males, with female-to-male ratios of 1.87:1 and 1.74:1, respectively. In addition, the burdens of ASMR and ASDR were also higher in females than in males, with female-to-male ratios of 1.91:1 and 1.77:1, respectively.

**Figure 4:**
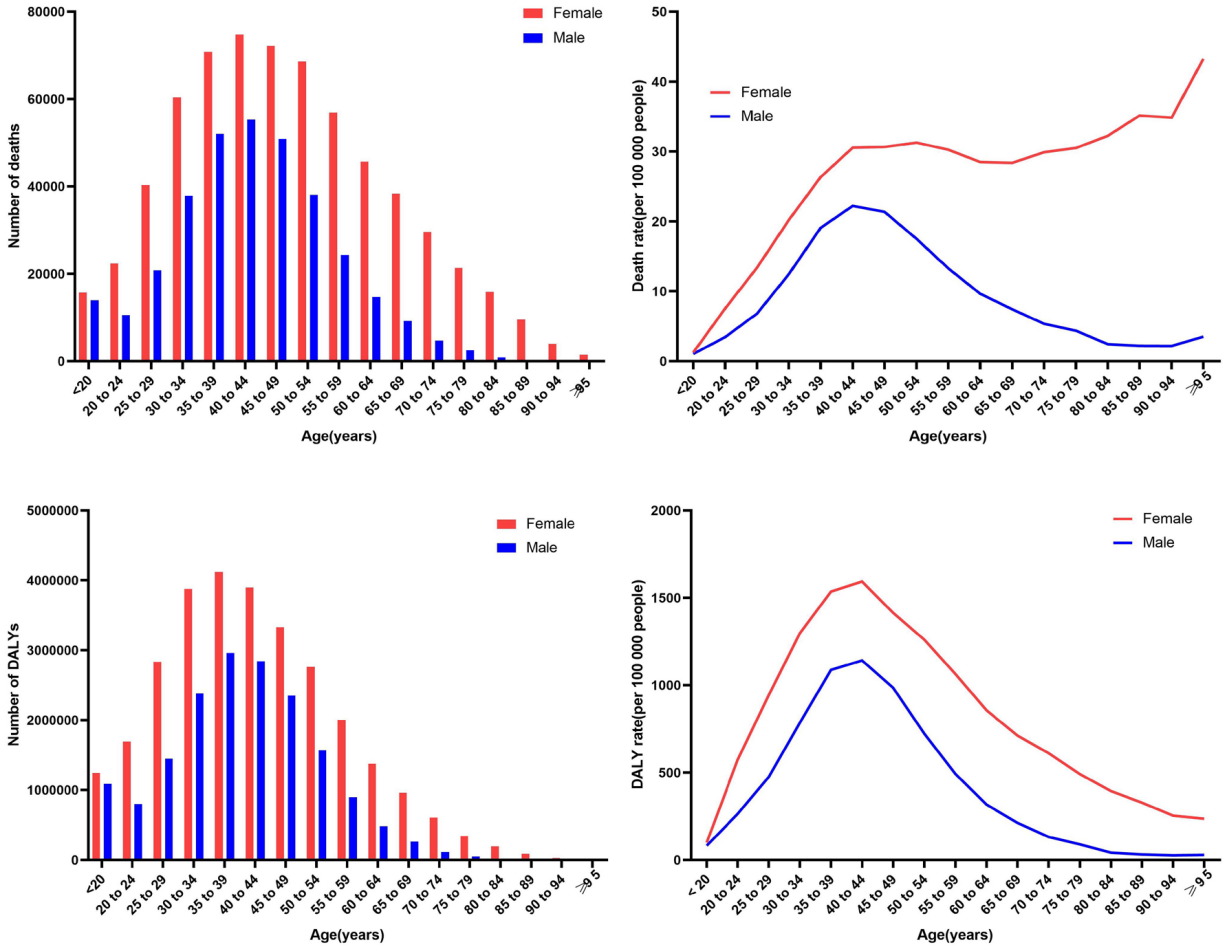
Age-specific numbers and rates of deaths and DALYs attributable to Unsafe Sex globally in 2019.

The highest population in unsafe sex-related deaths and DALYs worldwide was the 40–44 age group and 35–39 age group, respectively, for both men and women (Fig. 4). For women, the overall trend of global age-specific deaths attributed to unsafe sex increased rapidly for the population from the younger than 20 age group (16,000 deaths) to the 40–44 age group (75,000 deaths), remained at a plateau until the 65–69 age group (38,000 deaths), and then abruptly declined to the older than or equal to 95 age group (1500 deaths). The number of age-specific deaths attributed to unsafe sex had fallen significantly for men in comparison with that for women, with a rapid increase from those of the younger than 20 age group (14,000 deaths) to 40–44 age group (55,000 deaths) and then a slow decline to the older than or equal to 95 age group (45 deaths).

Interestingly, both the global ASMR and ASDR attributed to unsafe sex for males tended to increase precipitously from 1.05/100,000 people and 81.90/100,000 people, respectively, in the younger than 20 age group to 22.23/100,000 people and 1142.10/100,000 people, respectively, in the 40–44 age group and then decline to 4.37/100,000 people and 89.36/100,000 people, respectively, in the 75–79 age group. However, for females, the global death rate attributed to unsafe sex tended to increase swiftly from 1.26/100,000 people in the younger than 20 age group to the 40–44 age group of 30.55/100,000 people, stay in a plateau between the 45–49 age group (30.67/100,000 people) and the 65–69 age group (30.52/100,000 people), and then be a gradual uptrend, while the DALY rate increased rapidly from 99.61/100,000 people in the younger than 20 age group to 1593.36/100,000 people in the 40–44 age group, and then declined to 235.42/100,000 people in the 95 and older age group.

### Disease burden attributed to unsafe sex

As shown in Table 2, HIV/AIDS and sexually transmitted infections and neoplasms were the two GBD level 2 causes of ASMRs and ASDRs attributable to unsafe sex in 2019. The ASMRs of HIV/AIDS and sexually transmitted infections and neoplasms attributable to unsafe sex were 8.58 (95% UI: 7.72–10.05)/100,000 people and 3.40 (95% UI: 2.90–3.81)/100,000 people, respectively, and the ASDRs of HIV/AIDS and sexually transmitted infections and neoplasms attributable to unsafe sex were 463.58 (95% UI: 408.90–548.55)/100,000 people and 107.20 (95% UI: 90.52–119.43)/100,000 people, respectively. During the three decades between 1990 and 2019, the percentage change of ASMRs and ASDRs for HIV/AIDS and sexually transmitted infections increased by 74.45% (95% UI: 43.34–124.69%) in ASMR and 71.89% (95% UI: 44.43–110.60%) in ASDR, while those in Neoplasms decreased by − 23.95% (95% UI: − 35.20%, − 13.36%) in ASMR and − 23.42% (95% UI: − 35.05%, − 11.78%) in ASDR.

**Table 2:** Causes of deaths and DALYs attributable to unsafe sex for both sexes combined in 2019 and percentage change from 1990 to 2019.

Cause of death or DALYs	Deaths			DALYs	
	2019 age-standardized rate per 100,000 people	Percentage change in age-standardized rate, 1990–2019		2019 age-standardized rate per 100,000 people	Percentage change in age-standardized rate, 1990–2019
HIV/AIDS and sexually transmitted infections^**a**^	8.58 (7.72, 10.05)	74.45% (43.34%, 124.69%)		463.58 (408.90, 548.55)	71.89% (44.43%, 110.60%)
HIV/AIDS^**b**^	8.48 (7.62, 9.95)	79.69% (45.78%, 136.03%)		447.44 (394.82, 533.10)	78.98% (48.47%, 124.52%)
HIV/AIDS—drug-susceptible Tuberculosis^**c**^	1.99 (1.41, 2.61)	20.31% (− 9.92%, 74.02%)		103.24 (75.54, 134.47)	18.24% (− 9.92%, 64.74%)
HIV/AIDS—Multidrug-resistant Tuberculosis without extensive drug resistance^**c**^	0.19 (0.08, 0.36)	2019.49% (1080.51%, 3599.68%)		9.57 (4.10, 17.39)	1994.54% (1050.75%, 3555.31%)
HIV/AIDS—Extensively drug-resistant tuberculosis^**c**^	0.01 (0.00, 0.01)	–		0.32 (0.14, 0.59)	–
HIV/AIDS resulting in other diseases^**c**^	6.30 (5.26, 7.80)	105.86% (65.01%, 166.11%)		334.31 (279.95, 419.85)	106.07% (69.16%, 158.15%)
Sexually transmitted infections excluding HIV^**b**^	0.10 (0.08, 0.11)	− 49.73% (− 55.97%, − 40.92%)		16.14 (10.51, 25.83)	− 18.06% (− 27.11%, − 9.92%)
Syphilis^**c**^	0.02 (0.02, 0.03)	− 61.92% (− 67.73%, − 51.68%)		1.24 (0.95, 1.51)	− 51.20% (− 58.69%, − 39.42%)
Chlamydial infection^**c**^	0.01 (0.01, 0.01)	− 40.98% (− 49.35%, − 30.94%)		1.98 (1.41, 2.76)	− 15.68% (− 21.92%, − 9.56%)
Gonococcal infection^**c**^	0.04 (0.03, 0.04)	− 43.19% (− 50.85%, − 34.58%)		2.03 (1.65, 2.43)	− 34.12% (− 41.98%, − 25.22%)
Trichomoniasis^**c**^	–	–		–	4.89% (3.13%, 6.55%)
Genital herpes^**c**^	–	–		–	3.47% (2.04%, 4.81%)
Other sexually transmitted infections^**c**^	0.03 (0.02, 0.03)	− 47.35% (− 54.47%, − 38.09%)		4.28 (3.09, 5.78)	− 20.47% (− 26.66%, − 14.25%)
Neoplasms^**a**^	3.40 (2.90, 3.81)	− 23.95% (− 35.20%, − 13.36%)		107.20 (90.52, 119.43)	− 23.42% (− 35.05%, − 11.78%)
Cervical cancer^**b**^	3.40 (2.90, 3.81)	− 23.95% (− 35.20%, − 13.36%)		107.20 (90.52, 119.43)	− 23.42% (− 35.05%, − 11.78%)

^a^Cause groups at level 2.

^b^Cause groups at level 3.

^c^Cause groups at level 4.

**Figure 5:**
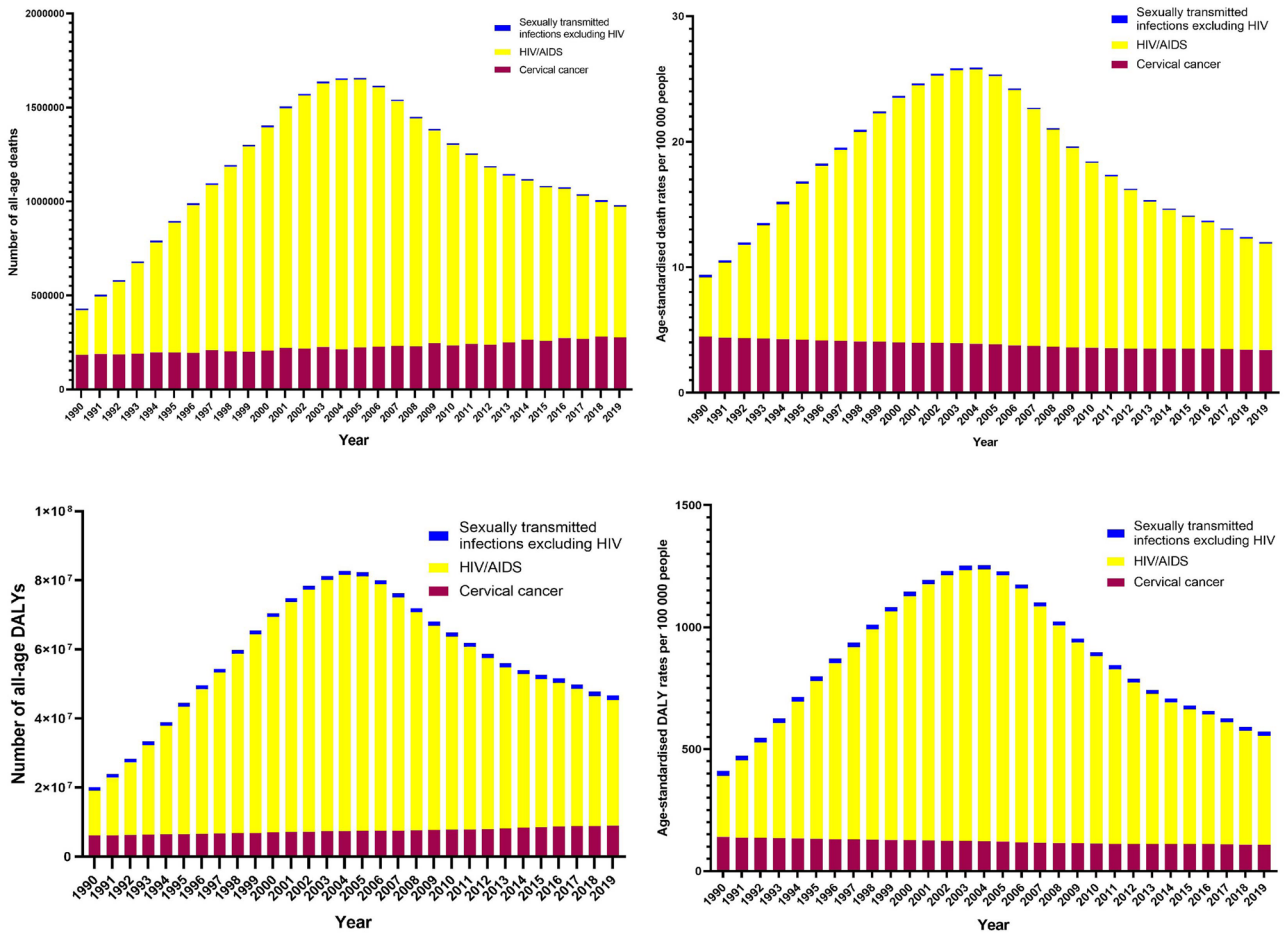
The numbers and rates of deaths and DALYs of level 3 diseases attributable to unsafe sex globally from 1990 to 2019.

Among the GBD grade 3 causes (Fig. 5), the disease unsafe-sex-related HIV/AIDS had the highest global ASMR [8.48 (95% UI: 7.62–9.95)/100,000 people] and ASDR [447.44 (95% UI: 394.82–533.10)/100,000 people] attributable to unsafe sex, followed by cervical cancer [ASMR: 3.40 (95% UI: 2.90–3.81)/100,000 people and ASDR: 107.2 (95% UI: 90.52–119.43)/100,000 people] and sexually transmitted infections excluding HIV [ASMR: 0.10 (95% UI: 0.08–0.11)/100,000 people and ASDR: 16.14 (95% UI: 10.51–25.83)/100,000 people]. From 1990 to 2019, the ASMRs and ASDRs of unsafe sex-related cervical cancer and sexually transmitted infections excluding HIV decreased by years, while unsafe sex-related HIV/AIDS increased precipitously in the first fourteen years and then decreased in the next fifteen years, with an absolute increase of 80% by 2019 compared to 1990. It was noteworthy that (Additional files 1 and 2), as a GBD grade 4 cause, the percentage change over the past 30 years of ASMR and ASDR caused by unsafe sex-related multidrug-resistant tuberculosis without extensive drug resistance was as high as 2019.49% (95% UI: 1080.51–3599.68%) and 1994.54% (95% UI: 1050.75–3555.31%), respectively.

### Disease burden attributed to unsafe sex in various age groups

**Figure 6:**
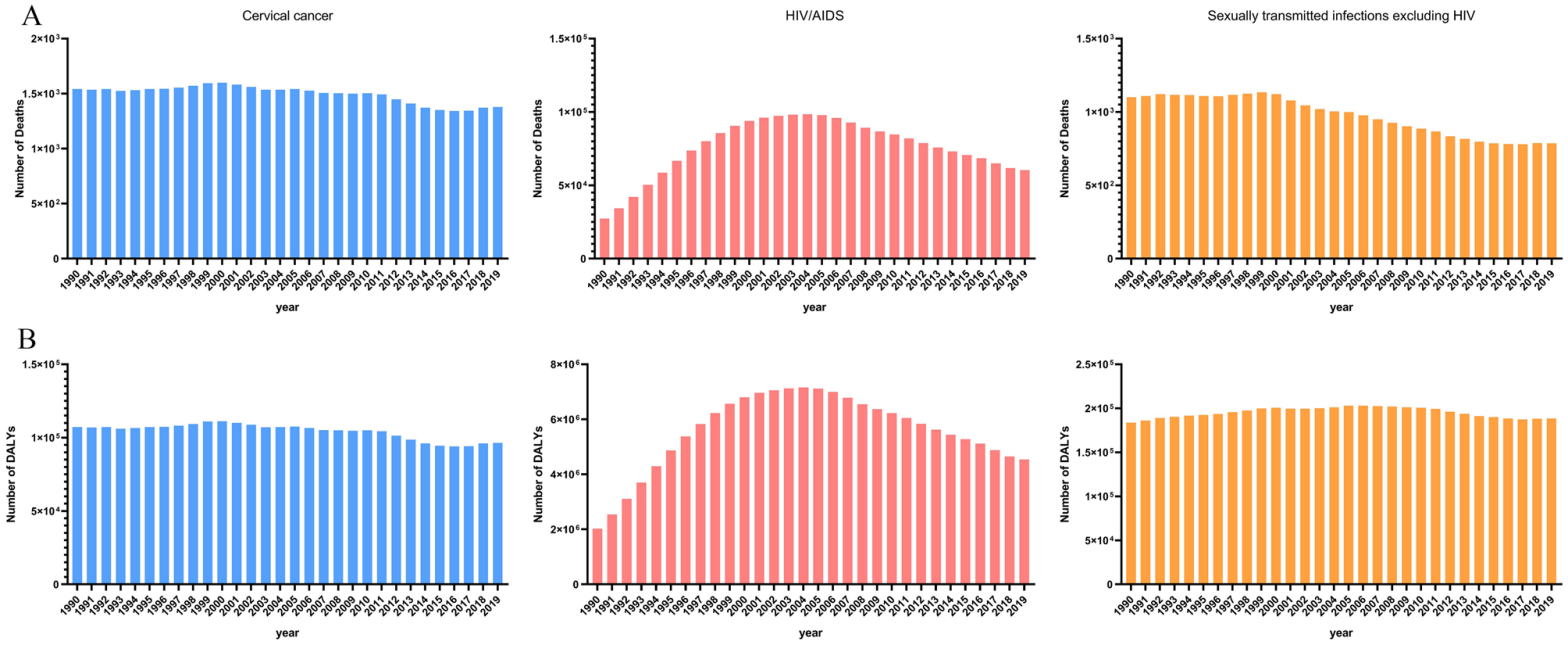
The numbers of deaths(A) and DALYs(B) of level 3 diseases attributable to unsafe sex in younger than 25 age group globally from 1990 to 2019.

**Figure 7:**
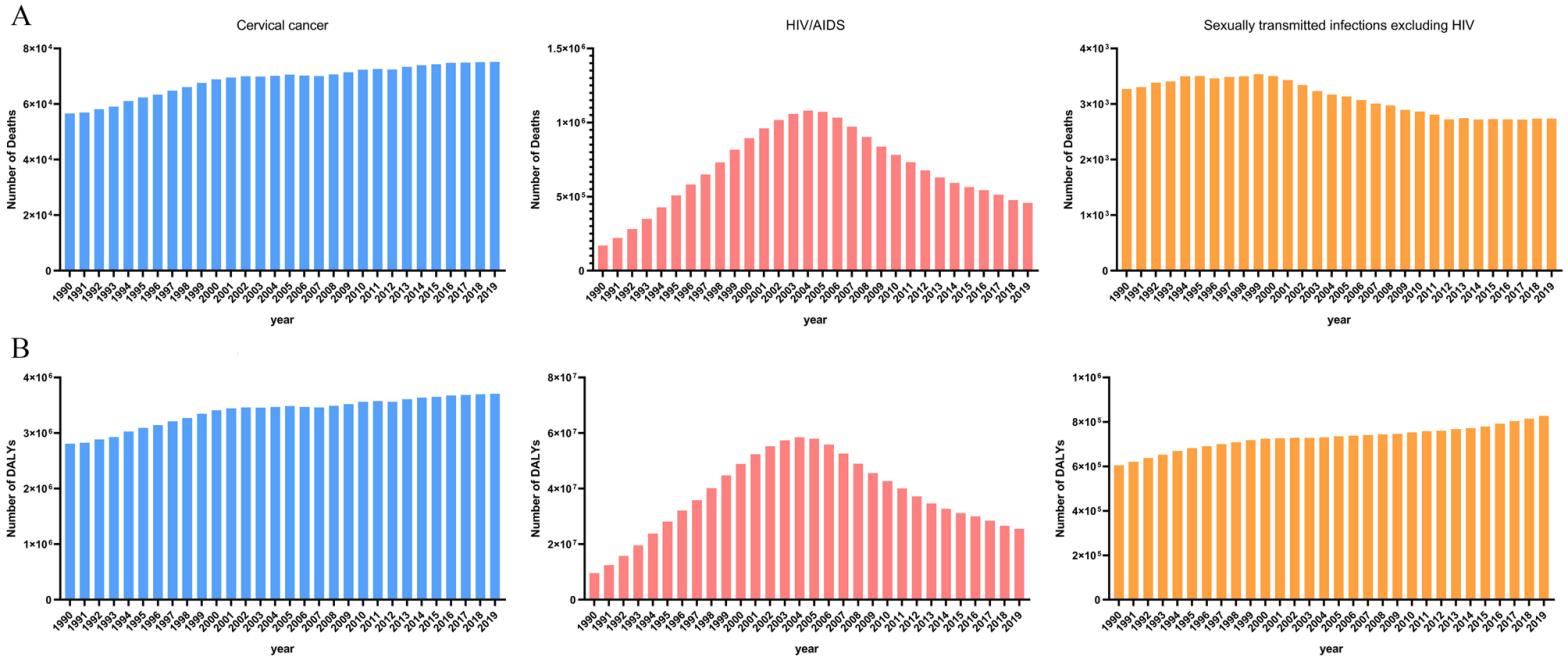
The numbers of deaths(A) and DALYs(B) of level 3 diseases attributable to unsafe sex in 25–49 age group globally from 1990 to 2019.

**Figure 8:**
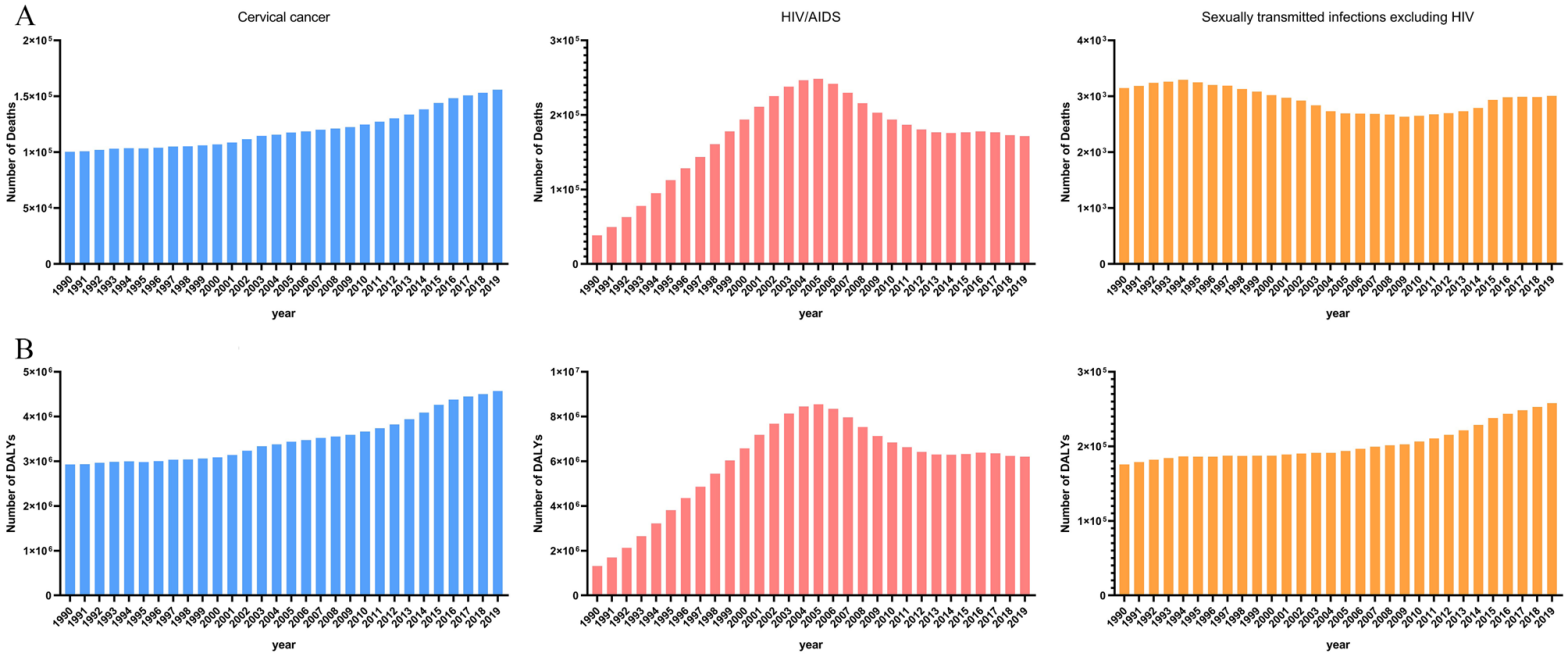
The numbers of deaths(A) and DALYs(B) of level 3 diseases attributable to unsafe sex in 50–74 age group globally from 1990 to 2019.

**Figure 9:**
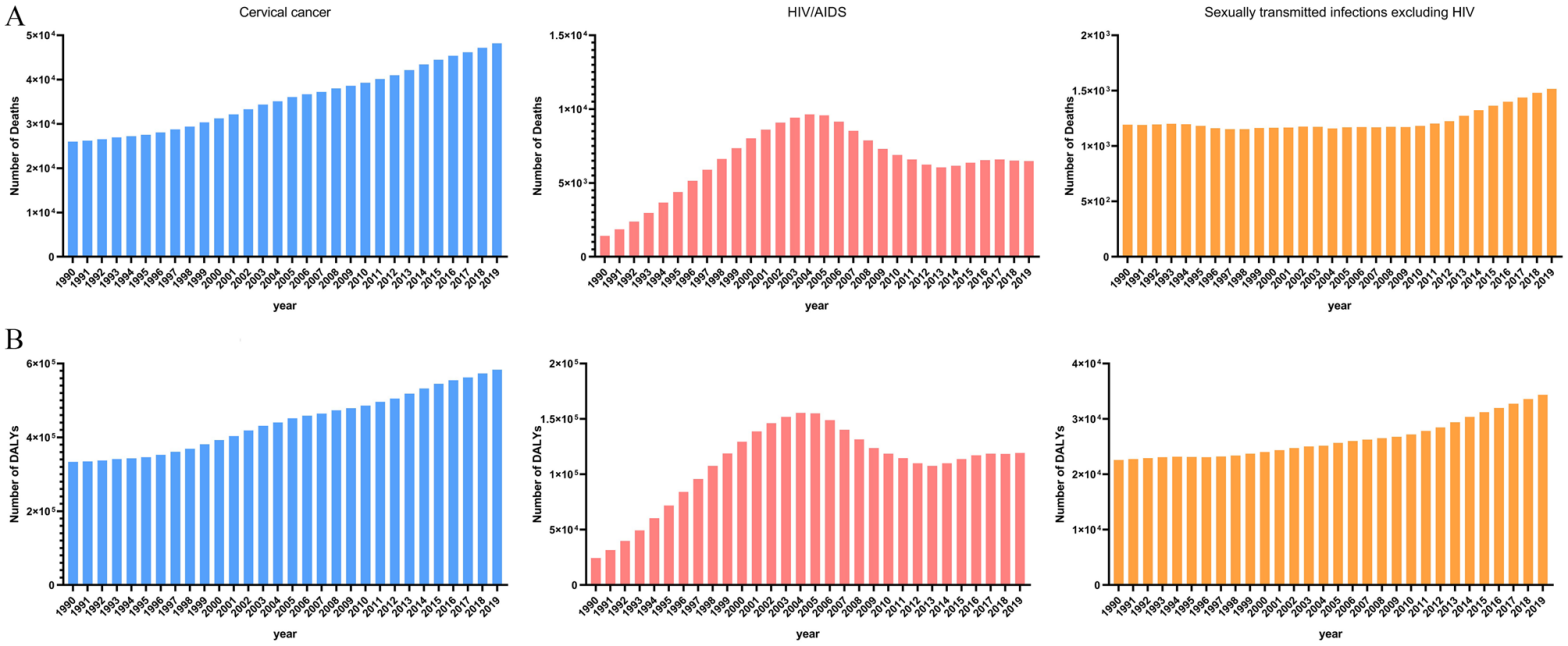
The numbers of deaths(A) and DALYs(B) of level 3 diseases attributable to unsafe sex in 75 and older age group globally from 1990 to 2019.

Figures 6, 7, 8 and 9 showed the results of a separate analysis of the burden attributable to unsafe sex in various age groups. Except for the group younger than 25 years old, the death and DALY burden of cervical cancer in other groups increased gradually, especially in the 75 and older age group. The burden trend of HIV/AIDS was similar to the overall trend, but the death and DALY burden of the 50–74 age group and 75 and older age group showed slow upward trends after 2014. Notably, the death burden in different age groups of sexually transmitted infections excluding HIV had decreased except for the 75 and older age group, and the burden of DALY had been increasing year by year except those under 25 years old.

### Relationship between SDI and the impact of unsafe sex on disease burden

**Figure 10:**
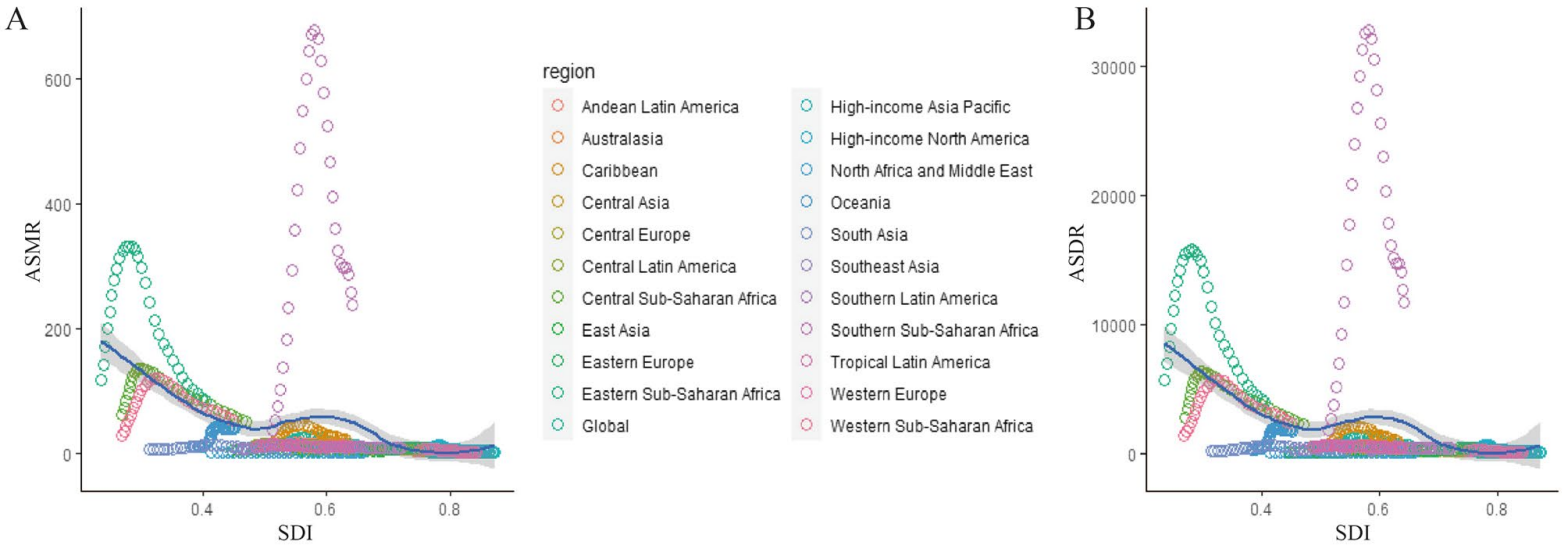
The ASMR (A) and ASDR (B) attributable to unsafe sex across 21 GBD regions by SDI for both sexes combined from 1990 to 2019. The blue line represents the average expected relationship between SDIs and ASRs for unsafe sex based on values from all countries from 1990 to 2019.

In regions with higher levels of SDI, both the ASMRs and ASDRs decreased (Fig. 10). While ASMRs and ASDRs remained stable in most areas from 1990 to 2019, they exhibited obvious fluctuations when the SDI value was less than 0.4, particularly in Eastern Sub-Saharan Africa, the SDI value of which was 0.235 and the ASMR of which was 117.26/100,000 people in 1990. With the SDI value and increasing years, the ASMR reached its peak of 331.44/100,000 people when the SDI value was 0.28 in 2001 and then decreased slowly.

**Figure 11:**
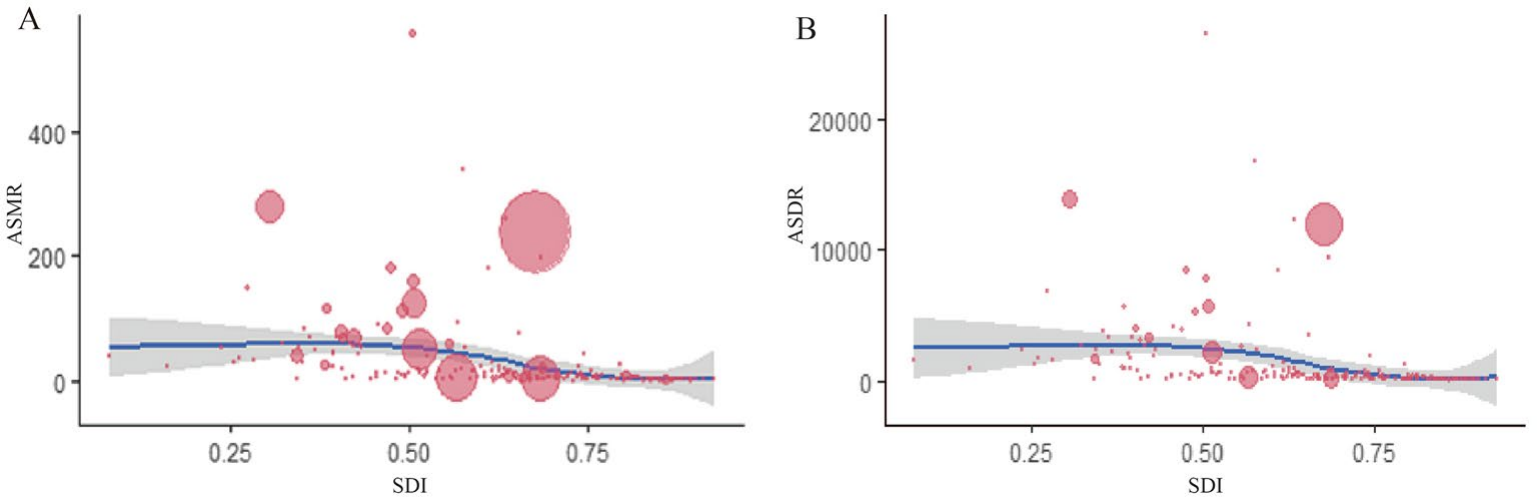
The correlation between ASMR (A) and ASDR (B) attributable to unsafe sex in 2019 and SDI, based on the values of ASR from all countries. The size of the circle is increased with the values of ASR, and one circle represents a specific country. The ρ indices and P value were derived from Pearson correlation analysis.

As shown in Fig. 11, in various countries, the increase in SDI value was associated with a gradual decrease in both ASMRs and ASDRs. Notably, Lesotho (559.40/100,000 people), Eswatini (340.59/100,000 people) and Mozambique (278.92/100,000 people) had much higher ASMRs than expected based on SDI values. Globally, deaths and DALYs caused by unsafe sex were concentrated in the age band of 25–49 years (Additional files 3 and 4); most notably, deaths and DALYs only tended to drop off over time in the high SDI regions rather than in the other quintile regions. The deaths and DALYs caused by unsafe sex-related cervical cancer or sexually transmitted infections excluding HIV showed an upward or gradual downward global tendency (Additional file 5).

## Discussion

Based on the GBD 2019 database, our results showed the basic situation of the disease burden caused by unsafe sex worldwide. Over the past three decades, the disease burden attributable to unsafe sex has increased in all regions of the world. In this study, our analysis demonstrated that global deaths attributable to unsafe sex rapidly increased from 430,000 new cases in 1990 to a peak of 1.7 million cases in 2004 and then slowly declined to 1 million cases in 2019. This study, to our knowledge, is the first to assess the deaths and DALYs attributable to unsafe sex. These findings can help formulate corresponding health service strategies and allocate health resources, as well as assess the effectiveness of current prevention and control measures. With these measures, the disease burden caused by unsafe sex is expected to gradually decrease in the future.

The number and ratio of mortality and DALYs, as well as ASMRs and ASDRs, are common indicators of disease burden^30^. In our study, both the global ASMR and ASDR attributed to unsafe sex for males tended to increase precipitously in the younger than 20 age group to a peak value in the 40–44 age group and then decline in the 75–79 age group. Due to the impact of global population growth^31^, the number of deaths and DALYs attributed to unsafe sex in countries and regions around the world increased by nearly 100% from 1990 to 2019. The demarcation points in 2004 suggested that the burden of unsafe sex was under management and control in 2004. The underlying reason could be attributed to the popularity of condoms, the developments in medical technology, and increased awareness of reproductive health^32,33^.

As a unique disease burden caused by unsafe sex, various STDs, e.g., HIV infection, always have potentially serious consequences^34,35^. HIV infection is widely recognized as the key prevention and control object of infectious disease^36^. With improved health education on unsafe sex and the promotion of sanitation and hygiene practices, the burden of HIV infection has decreased slowly since 2004. However, most unsafe sex-related HIV transmission occurs in patients without sexual safety; as a consequence, HIV prevention interventions for people living with HIV are still urgently required^20,37^. As previous studies have shown, when HIV viral load is suppressed, the risk of gay couples transmitting HIV through condom-free sex could be extremely low^38^. In addition, cervical cancer is the second most important disease that could be attributed to unsafe sex. From 1990 to 2019, the number of deaths and DALYs caused by unsafe sex-related cervical cancer showed an upward trend. However, the ASDR and ASDRs of unsafe sex-related cervical cancer have been declining, which is encouraging. The emergence of cervical cancer can be attributed to HPV infection caused by unsafe sex. Although the cervical cancer vaccine has begun to enter society, there is still a certain gap between universal access and universal access^39,40^. For example, in China, where the population is large, the HPV vaccine has not yet been included in the national immunization program due to its high cost or a huge vaccine gap in the market^41^. Overall, the prevention and control of unsafe sex would help reduce the risk of STDs worldwide.

The disease burden attributed to unsafe sex varies significantly across different countries and regions. In our study, the disease burden caused by unsafe sex was concentrated in sub-Saharan Africa, especially in the three countries Lesotho, Eswatini and South Africa, which have the most serious burden among the 204 countries in the world. Significantly, the disease burden of these three countries increased extremely high during the three decades from 1990 to 2019. Our results are consistent with the literature reporting that there are a large number of HIV carriers and AIDS-infected people in sub-Saharan Africa^42^. The main reasons for this are the prevalence of paying for sex in sub-Saharan Africa and the unwillingness to buy and use condoms, which are the only widely used low-cost HIV prevention technologies in South Africa^43–45^. Unfortunately, most HIV-positive patients in underdeveloped countries cannot have access to or opportunity to receive antiretroviral therapy or even conventional treatments for opportunistic infections^21,46,47^. Under the dual effects of sex trade and HIV infection, the disease burden caused by unsafe sex in sub-Saharan Africa increases and will continue to increase in the coming years. Therefore, in such a vicious social environment, national health authorities in sub-Saharan Africa should pay more attention to the prevention and control of the disease burden of unsafe sex, with the aim of overcoming a major obstacle hindering national or social development. In contrast, Australasia had the smallest disease burden due to the Australian government's significant investment in eliminating HIV infection^48,49^. Many states and local communities have or are currently considering banning the practice of taking off condoms during their sex life. Reasonable management of unsafe sex lowers the global burden of HIV infection and cervical cancer, and funds for monitoring epidemiological trends and assessing the progress of unsafe sex-related diseases are needed.

In our study, we found that the burden of unsafe sex also varies among SDI quartiles. In 2019, the burden of disease attributable to unsafe sex decreased with the increase in SDI quartile. Considering the inclusion of three variables in the SDI, this trend may be attributed to better social systems and advanced prevention and treatment technologies in high-SDI areas^25,50^. Unlike the cancer burden in 2017, the odds of developing cancer were the lowest in the low SDI quintile and the highest in the high SDI quintile for both sexes^51^. Unexpectedly, Southern Sub-Saharan Africa, a Middle-SDI quintile region with an increase in unsafe sex cases over the past three decades, had the highest ASMRs and ASDRs attributable to unsafe sex. Moreover, subjects in the 35–44 age group had relatively high ASMRs and ASDRs, and ASMRs in women showed an increasing trend with age. These results demonstrated that people of advanced age, especially women, should be given more attention. Disease prevention and treatment strategies should be adopted for the population.

The results of GBD 2019 Healthcare Access and Quality (HAQ) index showed that countries with higher SDI had better performance in the HAQ Index-nearly 50 points separated the lowest and highest SDI quintiles for the overall^52^. Promoting social and economic development was a fundamental measure to reduce the disease burden of unsafe sex. Social and economic development supported countries in mobilizing more funds and resources for projects, such as pooling resources for health insurance, improving health-care opportunities and quality^52^. Although low-income countries provided large amounts of funding each year to develop health assistance, they were still unable to keep up with the growing burden of disease. In low and low-middle income countries, there was growing evidence that simply expanding health care coverage did not necessarily lead to better results, even in circumstances that were very suitable for health care^53^. As a Middle-SDI quintile region, Sub-Saharan Africa had a low rate of acceptable mortality due to receipt of poor­ quality health services, where a greater percentage of mortality was non­utilization of services. For countries with high SDI to improve survival, medical availability was no longer the only constraint. The quality of healthcare must be improved at the same time.

This research also has some inevitable limitations. The accuracy of the research results depended on the quality of the data extracted from the GBD 2019 database. The data from GBD 2019 are collected from different databases and institutions through algorithms. Due to limited professional medical and treatment data in less developed countries, deviations between the data could not be excluded. Secondly, the database does not have a clear definition of unsafe sexual behavior, the statistics on relevant information may not be comprehensive, and it is impossible to conduct further classified research. Moreover, the database could not provide data related to exposure to unsafe sexual behavior. Therefore, we could not analyze the outcome caused by unsafe sex rather than the global exposure factors.

In short, our research used the GBD 2019 database to demonstrate the global disease burden attributable to unsafe sex, according to gender, age, country/region and social development. The global disease burden attributable to unsafe sex shows an overall downward trend. The results of this study may have important guiding significance in some countries and regions in the formulation of appropriate medical prevention and control policies and the allocation of medical resources, especially in southern sub-Saharan African areas. According to the World Health Organization’s strategies on HIV and sexually transmitted infections for 2022–2030, developing people-oriented integrated health services, providing more support for high-risk groups, eliminating vertical transmission, addressing children's care needs and reducing HIV-related mortality^54^. Continued global efforts are still needed to reduce the burden caused by unsafe sex.

## Conclusion

Unsafe sex is an important risk factor for global disease burden and is responsible for causing substantial health loss. We found that the risk of ASMRs and ASDRs attributable to unsafe sex is negatively correlated with SDI levels. The related diseases caused by unsafe sex, such as HIV/AIDS and cervical cancer, have the highest burden in southern sub-Saharan Africa and the lowest burden in Australasia. These results demonstrate that revised policies should concentrate on reducing overall unsafe sex worldwide.

### Supplementary Information

Supplementary Information 1.

Supplementary Information 2.

## Data Availability

The datasets supporting the conclusions of this article are included within the article and its additional files. All data were obtained from the public open database: Global Health Data Exchange (GHDx) query tool (https://ghdx.healthdata.org/gbd-results-tool).

## Abbreviations

DALYs: Disability-adjusted life years
GBD 2019: Global Disease Burden 2019
ASRs: Age-standardized rates
SDI: Social Demographic Index
ASMR: Age-standardized mortality rate
ASDR: Age-standardized DALY rate
HIV: Human immunodeficiency virus
AIDS: Acquired immunodeficiency syndrome
HPV: Human papillomavirus
STDs: Sexually transmitted diseases
GHDx: Global Health Data Exchange
DisMod-MR: Disease model—Bayesian meta-regression
YLDs: Years lived with disability
YLLs: Years of life lost
