# Supplementary material for: Global disease burden attributed to unsafe sex in 204 countries and territories from 1990 to 2019: results from the Global Burden of Disease Study 2019
Source: Sci Rep. 2023 Aug 9;13:12900. doi: 10.1038/s41598-023-40001-2 (PMC10412620; doi:10.1038/s41598-023-40001-2)
Supplement: Supplementary file 2 — Supplementary Information 2. [file 41598_2023_40001_MOESM2_ESM.pdf]

# Global disease burden attributed to unsafe sex in 204 countries and territories from 1990 to 2019: results from the Global Burden of Disease Study 2019

**Pei Qiu[1,2, #], Hairong He[3, #], Yuting Zhao[1,2], Zejian Yang[1,2], Shouyu Li[1,2], Peng Ni[1,2], Yujie Guo[2], Chao Ji[2], Chenchen Zhang[4], Huimin Zhang[1], Can Zhou[1, *] and Bo Wang[5,6, **]**

[1]Department of Breast Surgery, The First Affiliated Hospital of Xi'an Jiaotong University, Xi'an, Shaanxi, China.

[2]School of Medicine, Xi'an Jiaotong University, Xi'an, Shaanxi, China

[3]Clinical Research Center, The First Affiliated Hospital of Xi'an Jiaotong University, Xi'an, Shaanxi, China.

[4]Department of Clinical Laboratory, the 940th Hospital of Joint Logistics Support Force of Chinese People's Liberation Army, Lanzhou, China

[5]Center for Translational Medicine, the First Affiliated Hospital of Xi'an Jiaotong University, Xi'an, China;

[6]Key Laboratory for Tumor Precision Medicine of Shaanxi Province, Xi'an, China.

# Pei Qiu and Hairong He contributed equally to this work and share first authorship.

*Corresponding author: Can Zhou, Address: 277 Yanta Western Rd., Xi’an, 710061, Shaan’xi Province, China. Tel: (+86) 029-85324605. Email: zhoucanz2005@xjtufh.edu.cn;

**Corresponding author: Bo Wang, Address: 277 Yanta Western Rd., Xi’an, 710061, Shaan’xi Province, China. Tel: (+86) 029-85324605. Email: realwbo@xjtu.edu.cn

**Additional file 1.** The numbers (A) and ASRs (B) of deaths and DALYs of level 4 diseases under HIVAIDS attributable to unsafe sex globally from 1990 to 2019

**Additional file 2.** The numbers (A) and ASRs (B) of deaths and DALYs of level 4 diseases under sexually transmitted infections excluding HIV attributable to unsafe sex globally from 1990 to 2019

**Additional file 3.** The deaths of the four age groups from 1990 to 2019 globally and in the high, high-middle, middle, low-middle, and low SDI quintiles

**Additional file 4.** The DALY cases of the four age groups from 1990 to 2019 globally and in the high, high-middle, middle, low-middle, and low SDI quintiles.

**Additional file 5.** The death (A) and DALYs (B) of the three level 3 causes attributable to unsafe sex from 1990 to 2019

**Table 1.** Global, regional and national ASRs of deaths and DALYs attributable to unsafe sex in 2019 and percentage changes from 1990 to 2019. The bold text means 21 GBD regions.

**Table 2.** Causes of deaths and DALYs attributable to unsafe sex for both sexes combined in 2019 and percentage change from 1990 to 2019.

**Additional file 1.**

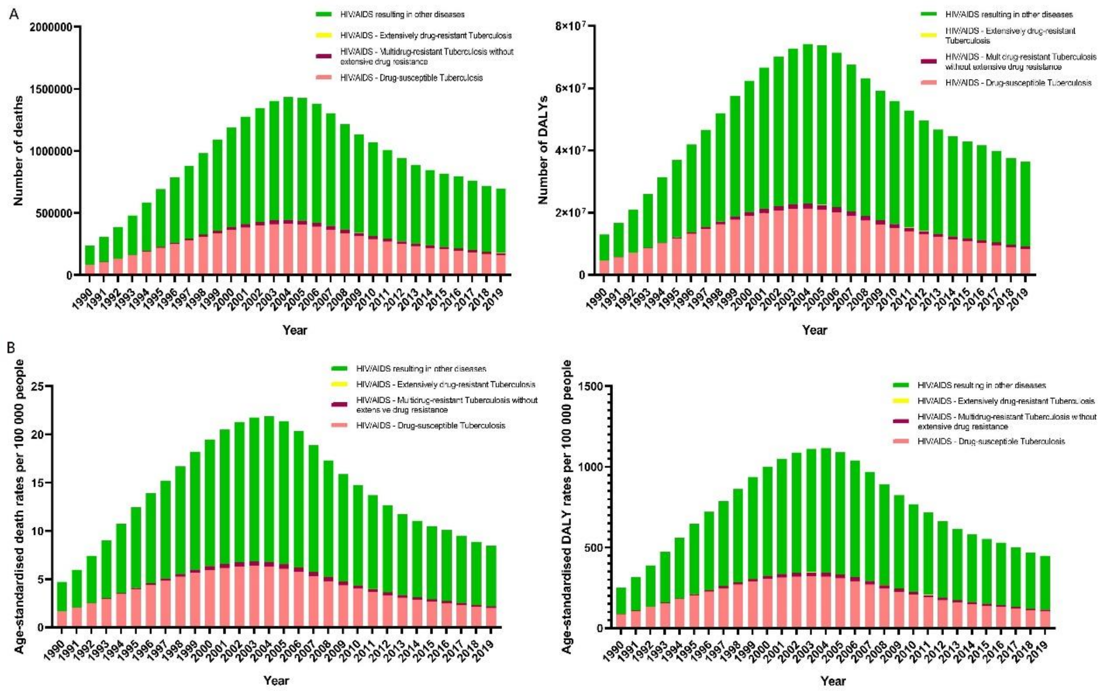

# Additional file 2.

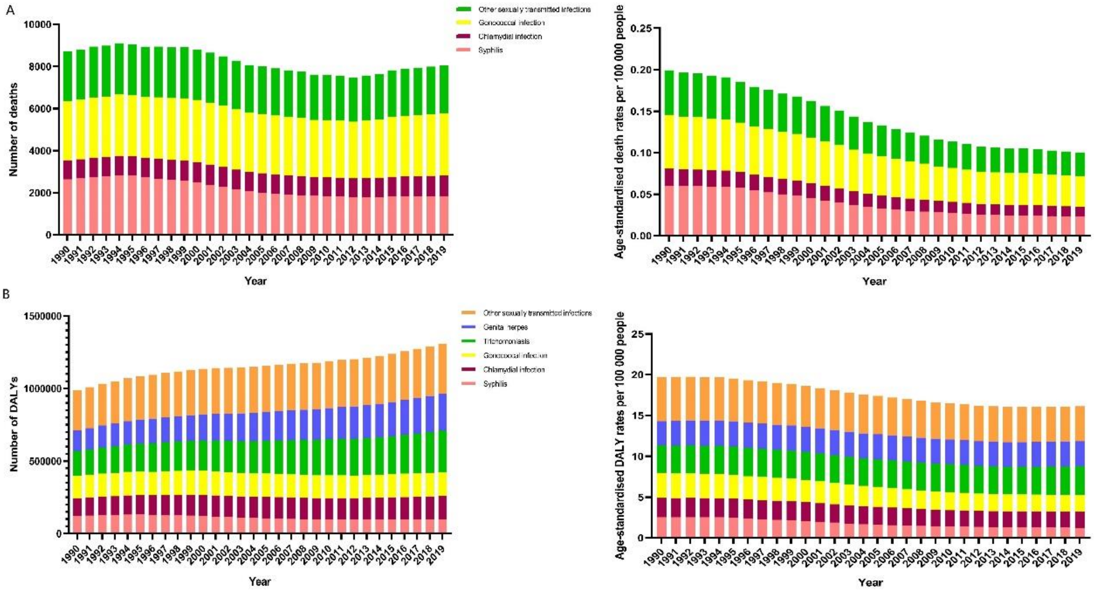

**Additional file 3.**

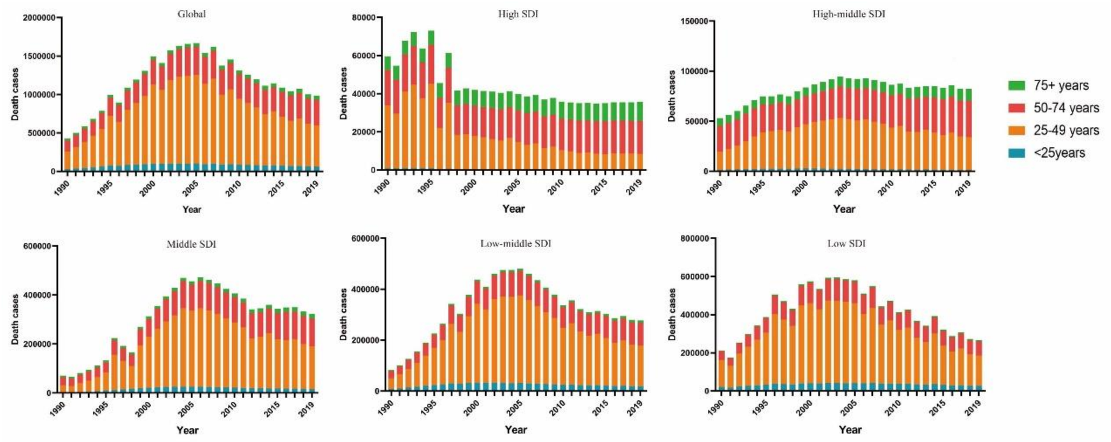

## Additional file 4.

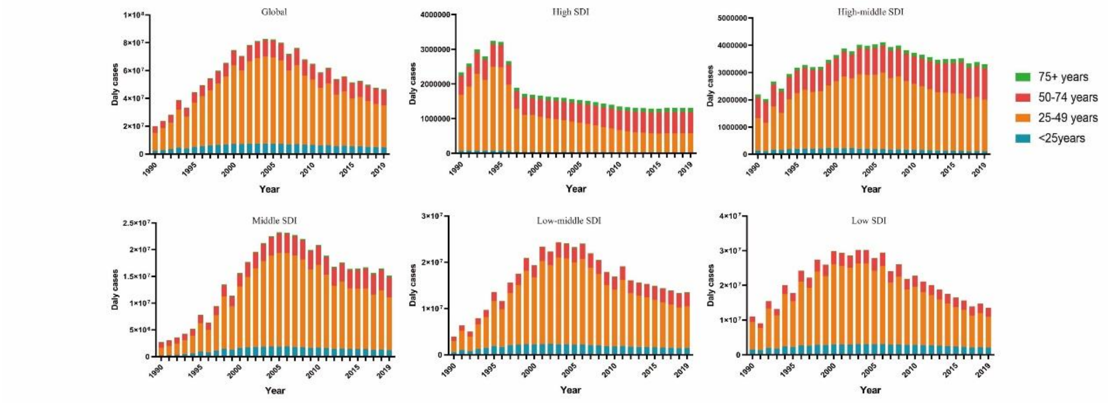

# Additional file 5.

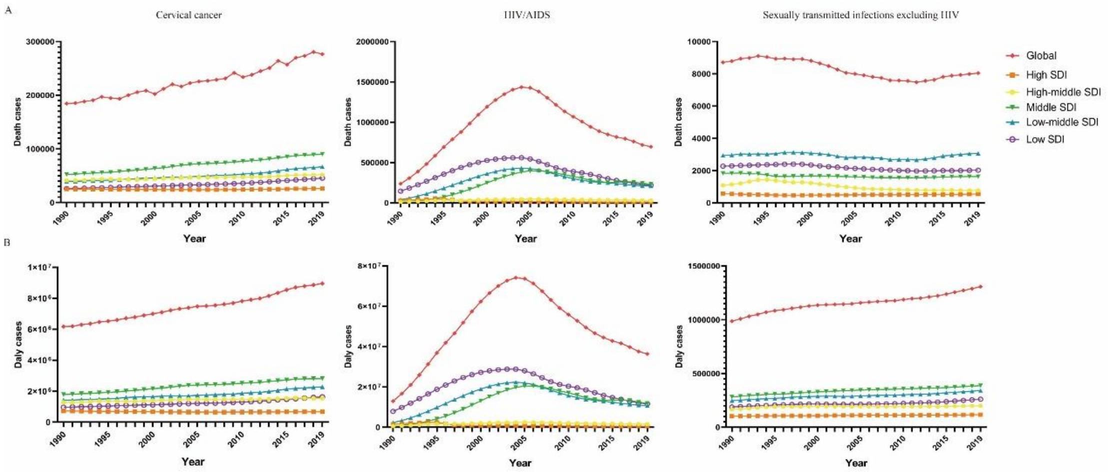

**Table 1.**

| | Deaths | | DALYs | |
|---|---|---|---|---|
| | 2019 age-standardized rate per 100,000 people | Percentage change in age-standardized rate,1990-2019 | 2019 age-standardized rate per 100,000 people | Percentage change in age-standardized rate,1990-2019 |
| Global | 11.98(10.97,13.52) | 27.64%(13.89%,44.60%) | 570.78(510.24,658.10) | 39.33%(25.14%,58.37%) |
| Low-SDI quintile | 34.33(31.13,38.55) | -29.40%(-44.35%, -5.58%) | 1579.77(1410.49,1829.90) | -31.68%(-45.33%, -10.23%) |
| Low-middle-SDI quintile | 16.95(15.59,18.95) | 61.57%(34.32%,91.24%) | 776.91(700.54,883.35) | 75.25%(44.20%,107.83%) |
| Middle-SDI quintile | 12.22(11.03,13.95) | 116.28%(75.02%,146.32%) | 567.12(508.55,660.33) | 183.31%(134.83%,222.39%) |
| High-middle-SDI quintile | 4.35(3.82,4.75) | -9.65%(-24.63%, -0.70%) | 184.40(164.50,202.50) | 5.04%(-11.12%,14.07%) |
| High-SDI quintile | 2.24(2.06,2.36) | -60.89%(-63.23%, -58.52%) | 96.88(85.99,110.93) | -61.46%(-64.93%, -57.23%) |
| **Central Sub-Saharan Africa** | **49.85(42.17,59.17)** | **-18.01%(-37.84%,8.05%)** | **2207.76(1850.96,2672.43)** | **-21.23%(-39.70%,3.82%)** |
| Angola | 83.76(61.50,114.63) | 400.07%(226.02%,659.97%) | 3882.27(2778.72,5417.95) | 532.69%(310.70%,863.89%) |
| Central African Republic | 150.41(124.30,190.61) | 81.81%(17.41%,155.97%) | 6834.70(5559.11,8946.15) | 64.37%(7.59%,127.90%) |
| Congo | 94.41(80.12,112.77) | -37.57%(-57.54%, -7.58%) | 4336.97(3638.97,5222.58) | -38.92%(-58.17%, -10.95%) |
| Democratic Republic of the Congo | 26.11(20.29,32.83) | -60.95%(-73.15%, -40.53%) | 1063.49(849.23,1348.81) | -65.75%(-76.13%, -47.48%) |
| Equatorial Guinea | 197.03(129.29,305.77) | 797.25%(421.16%,1436.93%) | 9443.61(6082.09,14869.52) | 1000.00%(514.75%,1810.18%) |
| Gabon | 76.37(57.05,105.11) | 162.89%(39.92%,333.53%) | 3488.60(2576.52,4952.74) | 180.89%(31.85%,397.45%) |
| **Eastern Sub-Saharan Africa** | **83.36(76.74,93.07)** | **-28.90%(-45.36%,2.36%)** | **3911.17(3554.46,4506.58)** | **-31.43%(-45.99%, -5.35%)** |
| Burundi | 34.44(27.52,43.09) | -75.21%(-90.44%, -47.85%) | 1469.14(1199.24,1789.34) | -78.36%(-91.45%, -54.79%) |

| | | | | |
|---|---|---|---|---|
| Comoros | 12.40(8.17,18.53) | -13.88%(-41.73%,70.23%) | 420.81(273.47,624.63) | -15.97%(-45.07%,84.12%) |
| Djibouti | 89.88(67.24,122.63) | 573.68%(335.37%,1071.98%) | 4035.99(2954.14,5674.65) | 795.31%(464.61%,1470.36%) |
| Eritrea | 42.90(33.49,53.56) | 22.06%(-27.07%,94.07%) | 1735.93(1330.68,2185.94) | 15.41%(-34.23%,84.09%) |
| Ethiopia | 38.73(33.41,45.83) | -4.52%(-38.88%,44.83%) | 1697.87(1450.16,2000.08) | -10.16%(-41.83%,34.23%) |
| Kenya | 123.01(109.29,139.35) | 29.91%(-18.46%,113.99%) | 5656.34(5001.53,6409.05) | 17.22%(-22.90%,80.77%) |
| Madagascar | 22.94(17.54,29.54) | 64.80%(22.68%,128.52%) | 946.06(725.18,1231.84) | 80.94%(32.04%,155.17%) |
| Malawi | 117.62(102.97,137.34) | -32.41%(-57.26%,10.99%) | 5669.32(4942.01,6691.23) | -35.32%(-57.87%,2.09%) |
| Mozambique | 278.92(232.79,357.85) | 818.37%(577.94%,1143.18%) | 13916.50(11468.42,18127.08) | 903.32%(629.37%,1223.18%) |
| Rwanda | 43.49(37.19,51.03) | -14.31%(-57.35%,39.67%) | 1890.90(1628.31,2221.61) | -18.68%(-60.00%,33.57%) |
| Somalia | 38.61(29.06,50.63) | 95.49%(36.29%,189.71%) | 1557.02(1203.75,1998.51) | 121.40%(55.05%,225.87%) |
| South Sudan | 71.26(35.83,134.10) | 354.58%(108.52%,860.11%) | 3344.80(1605.88,6592.53) | 434.91%(123.05%,1082.61%) |
| United Republic of Tanzania | 71.64(61.10,84.34) | -54.07%(-68.30%, -29.06%) | 3344.67(2864.06,4009.68) | -56.29%(-69.21%, -35.33%) |
| Uganda | 81.73(69.49,99.48) | -85.02%(-88.13%, -77.84%) | 3978.66(3310.24,4955.23) | -84.90%(-87.78%, -78.82%) |
| Zambia | 162.74(142.18,189.39) | -12.66%(-42.98%,41.99%) | 7857.36(6866.24,9262.60) | -17.09%(-45.85%,26.06%) |
| **Southern Sub-Saharan Africa** | **237.92(212.64,279.30)** | **542.00%(283.30%,855.70%)** | **11711.17(10265.58,14089.39)** | **538.35%(284.94%,820.69%)** |
| Botswana | 261.71(222.49,323.75) | 147.74%(55.40%,276.97%) | 12284.24(10323.94,15285.87) | 123.51%(47.08%,222.73%) |
| Lesotho | 559.40(480.85,682.46) | 1308.37%(869.35%,1828.26%) | 26530.50(22530.83,32807.37) | 1227.93%(832.65%,1653.43%) |
| Namibia | 180.09(156.44,212.96) | 589.01%(375.53%,916.19%) | 8441.76(7350.01,9882.42) | 548.20%(365.20%,784.93%) |
| South Africa | 240.15(210.62,289.65) | 1406.98%(1091.33%,1845.29%) | 11987.64(10295.82,14664.28) | 1556.90%(1285.78%,1918.88%) |

| | | | | |
|---|---|---|---|---|
| Eswatini | 340.59(297.11,402.25) | 1947.93%(1338.16%,3019.66%) | 16728.30(14591.41,19923.44) | 2413.93%(1675.26%,3517.61%) |
| Zimbabwe | 181.34(164.84,202.64) | 42.35%(-37.72%,172.44%) | 8437.46(7680.40,9346.22) | 30.45%(-41.95%,144.32%) |
| **Western Sub-Saharan Africa** | **52.17(46.39,59.27)** | **80.10%(34.20%,129.21%)** | **2331.39(2042.49,2721.33)** | **76.87%(29.67%,124.78%)** |
| Benin | 30.98(25.27,37.84) | 139.87%(80.89%,207.73%) | 1299.34(1041.43,1633.62) | 185.26%(17.86%,267.77%) |
| Burkina Faso | 28.53(23.76,33.93) | -82.63%(-88.04%, -73.01%) | 1161.43(972.29,1399.04) | -85.32%(-89.78%, -77.78%) |
| Cameroon | 115.16(102.99,133.11) | 376.73%(262.12%,511.13%) | 5239.84(4643.06,6158.28) | 394.38%(276.12%,533.88%) |
| Cabo Verde | 16.61(11.60,27.68) | -15.31%(-51.59%,54.91%) | 643.05(414.71,1218.40) | -18.83%(-57.64%,72.81%) |
| Chad | 53.83(41.02,71.86) | 110.57%(33.81%,201.36%) | 2387.61(1767.55,3269.09) | 113.68%(31.85%,214.04%) |
| Cote d'Ivoire | 69.41(58.89,80.67) | -22.48%(-69.68%,53.57%) | 3089.00(2623.77,3656.46) | -30.19%(-72.52%,39.66%) |
| The Gambia | 71.19(49.90,98.27) | 623.06%(376.69%,960.76%) | 3270.76(2269.69,4637.00) | 736.91%(460.94%,1116.21%) |
| Ghana | 60.86(52.52,71.98) | 108.42%(58.26%,172.52%) | 2740.17(2315.54,3335.45) | 110.04%(59.85%,174.93%) |
| Guinea | 60.46(46.91,77.11) | 134.21%(74.33%,214.87%) | 2665.44(2014.63,3532.42) | 176.70%(103.17%,276.43%) |
| Guinea-Bissau | 83.79(53.75,129.15) | 262.46%(124.14%,472.46%) | 3797.83(2395.76,5973.21) | 302.17%(149.62%,538.01%) |
| Liberia | 49.99(39.72,62.67) | 170.77%(76.53%,294.27%) | 2206.10(1750.78,2830.13) | 192.31%(82.24%,353.24%) |
| Mali | 37.79(30.15,46.63) | 93.08%(19.96%,178.46%) | 1675.86(1302.48,2103.45) | 101.66%(16.53%,197.63%) |
| Mauritania | 9.78(6.71,15.05) | -45.82%(-73.43%, -11.19%) | 329.37(222.28,546.82) | -49.23%(-75.45%, -11.99%) |
| Niger | 22.29(17.51,27.56) | 31.24%(-4.49%,79.59%) | 863.32(665.74,1070.23) | 30.06%(-7.00%,80.32%) |
| Nigeria | 49.02(40.15,59.14) | 233.21%(164.18%,315.36%) | 2213.38(1784.24,2733.04) | 243.51%(172.48%,324.13%) |
| Sao Tome and Principe | 13.84(9.59,18.82) | -7.95%(-31.10%,32.15%) | 443.90(319.45,594.56) | -12.26%(-35.81%,28.11%) |

| | | | | |
|---|---|---|---|---|
| Senegal | 23.56(19.00,28.63) | 56.60%(19.70%,103.53%) | 950.80(764.56,1167.08) | 61.78%(22.41%,111.11%) |
| Sierra Leone | 52.58(42.97,66.98) | 292.84%(174.40%,461.21%) | 2389.44(1914.80,3093.87) | 357.30%(203.86%,581.38%) |
| Togo | 61.50(48.42,77.04) | 174.47%(79.42%,286.38%) | 2704.02(2107.92,3469.07) | 187.25%(78.67%,317.87%) |
| **Andean Latin America** | **12.70(10.24,16.85)** | **1.58%(-20.54%,37.56%)** | **503.85(401.14,708.30)** | **14.54%(-14.30%,61.33%)** |
| Bolivia | 17.56(10.62,38.62) | -17.28%(-53.86%,87.16%) | 615.32(324.23,1677.97) | -14.05%(-59.28%,129.57%) |
| Ecuador | 13.34(15.56,11.64) | 37.89%(20.45%,61.69%) | 545.38(488.23,621.86) | 66.98%(48.36%,92.97%) |
| Peru | 11.10(8.30,14.31) | -2.81%(-27.48%,31.82%) | 451.98(337.91,603.38) | 8.89%(-19.75%,51.86%) |
| **Tropical Latin America** | **9.50(8.88,10.46)** | **-16.20%(-20.73%, -11.50%)** | **406.91(375.23,443.82)** | **-8.64%(-13.50%, -3.44%)** |
| Brazil | 9.39(8.78,10.39) | -17.30%(-21.86%, -12.51%) | 403.64(372.54,440.21) | -9.79%(-14.52%, -4.58%) |
| Paraguay | 13.60(10.18,17.38) | 32.09%(-0.83%,72.96%) | 532.45(404.17,670.43) | 46.96%(9.72%,92.70%) |
| **Central Latin America** | **10.04(9.04,11.34)** | **-23.76%(-31.97%, -12.26%)** | **409.15(373.42,454.98)** | **-10.78%(-18.60%, -1.08%)** |
| Colombia | 9.04(7.91,10.37) | -9.67%(-21.85%,4.56%) | 384.76(342.37,433.00) | 8.43%(-4.01%,22.65%) |
| Costa Rica | 6.50(5.56,7.79) | -32.61%(-43.04%, -15.58%) | 265.23(231.41,310.18) | -22.49%(-32.27%, -7.22%) |
| El Salvador | 18.32(13.26,23.37) | 67.42%(18.91%,119.76%) | 730.34(531.30,918.02) | 82.99%(30.74%,137.41%) |
| Guatemala | 14.09(10.88,17.13) | 18.40%(-34.31%,48.08%) | 520.55(427.45,617.15) | 13.04%(-29.68%,39.20%) |
| Honduras | 6.94(4.81,9.88) | -13.14%(-35.80%,17.47%) | 258.08(185.09,362.04) | -22.07%(-41.18%,4.02%) |
| Mexico | 8.72(7.71,10.40) | -41.87%(-49.66%, -17.41%) | 349.75(314.77,404.93) | -30.49%(-37.67%, -10.60%) |
| Nicaragua | 17.02(13.13,21.63) | 41.03%(6.68%,88.13%) | 657.71(494.23,831.97) | 56.89%(17.83%,107.48%) |
| Panama | 17.03(15.62,18.64) | 1.29%(-8.99%,16.47%) | 806.03(744.65,871.28) | 17.36%(7.13%,33.09%) |

| | | | | |
|---|---|---|---|---|
| Venezuela | 13.70(11.55,16.30) | 1.82%(-15.67%,23.26%) | 568.84(489.92,666.26) | 15.72%(-1.87%,37.28%) |
| **Southern Latin America** | **8.28(7.50,8.78)** | **2.71%(-8.13%,10.04%)** | **349.46(318.69,385.34)** | **15.79%(5.65%,27.09%)** |
| Argentina | 9.22(8.00,9.85) | 24.34%(0.00%,35.47%) | 394.38(347.38,438.26) | 34.49%(14.88%,49.59%) |
| Chile | 6.22(5.80,6.78) | -41.56%(-46.12%, -28.72%) | 252.53(231.10,287.06) | -28.40%(-34.45%, -15.28%) |
| Uruguay | 7.93(7.24,8.57) | 12.75%(1.23%,23.39%) | 314.10(287.15,343.50) | 24.67%(13.30%,35.32%) |
| **Caribbean** | **20.97(18.01,24.39)** | **14.41%(-8.90%,37.02%)** | **937.98(797.21,1114.91)** | **16.72%(-7.76%,42.45%)** |
| Antigua and Barbuda | 11.32(10.17,12.50) | -32.48%(-39.09%, -24.66%) | 447.64(404.58,490.21) | -34.03%(-39.26%, -27.75%) |
| The Bahamas | 26.59(24.44,28.54) | -6.75%(-12.33%,0.05%) | 1203.30(1093.39,1307.07) | -6.50%(-11.48%, -1.14%) |
| Barbados | 12.71(11.23,14.36) | -40.86%(-47.24%, -33.60%) | 503.67(449.49,562.57) | -40.96%(-46.39%, -34.65%) |
| Belize | 24.29(22.38,26.22) | -2.66%(-9.66%,5.13%) | 1081.54(991.21,1173.07) | 7.33%(0.51%,14.45%) |
| Bermuda | 7.70(7.17,8.31) | -63.96%(-65.93%, -61.35%) | 344.31(316.48,372.73) | -61.61%(-63.51%, -59.29%) |
| Cuba | 6.06(5.15,7.04) | 2.18%(-12.64%,19.85%) | 251.55(217.56,292.28) | 15.31%(0.74%,32.51%) |
| Dominica | 14.12(12.09,16.48) | -29.65%(-39.36%, -17.26%) | 549.13(478.58,627.98) | -27.31%(-36.43%, -16.75%) |
| Dominican Republic | 17.25(13.69,22.52) | 69.30%(15.75%,130.69%) | 703.90(545.60,951.56) | 68.32%(11.12%,135.21%) |
| Grenada | 12.77(11.20,14.12) | -37.72%(-45.37%, -29.84%) | 467.71(413.68,517.51) | -40.15%(-47.10%, -32.78%) |
| Guyana | 29.29(25.99,32.94) | 16.11%(1.19%,33.08%) | 1312.11(1168.71,1469.14) | 37.24%(21.42%,55.27%) |
| Haiti | 56.71(45.81,69.04) | 4.06%(-30.49%,49.43%) | 2409.72(1934.29,2994.41) | -0.53%(-35.29%,47.03%) |
| Jamaica | 19.12(16.80,21.69) | 15.75%(3.30%,30.80%) | 798.91(708.16,892.18) | 24.17%(13.06%,37.79%) |
| Puerto Rico | 6.44(5.84,7.15) | -71.99%(-74.54%, -68.65%) | 285.88(257.12,316.30) | -75.03%(-77.22%, -72.42%) |

| | | | | |
|---|---|---|---|---|
| Saint Lucia | 10.00(8.69,11.58) | -47.49%(-54.34%, -39.44%) | 379.60(333.98,433.38) | -45.62%(-52.08%, -37.70%) |
| Saint Vincent and the Grenadines | 22.77(20.83,24.77) | -22.65%(-29.14%, -15.61%) | 950.42(866.00,1032.73) | -15.36%(-21.54%, -8.06%) |
| Suriname | 21.56(19.30,24.08) | -9.24%(-17.71%,0.48%) | 930.69(841.42,1022.52) | -2.59%(-10.16%,6.00%) |
| Trinidad and Tobago | 15.81(14.03,17.95) | -4.54%(-14.90%,8.33%) | 692.75(620.16,775.76) | 10.22%(0.47%,21.96%) |
| United States Virgin Islands | 9.62(8.53,10.87) | -21.09%(-30.42%, -10.01%) | 383.58(343.31,425.52) | -18.55%(-26.92%, -8.82%) |
| **Central Europe** | **3.83(3.26,4.40)** | **-33.90%(-43.45%, -24.36%)** | **131.58(112.22,150.40)** | **-32.55%(-41.47%, -23.22%)** |
| Albania | 1.70(1.22,2.33) | -16.64%(-41.34%,14.83%) | 62.12(45.61,82.82) | -16.19%(-37.25%,11.47%) |
| Bosnia and Herzegovina | 3.06(2.15,3.94) | -15.13%(-37.44%,11.18%) | 103.95(74.08,133.18) | -12.14%(-35.45%,14.37%) |
| Bulgaria | 4.63(3.33,5.96) | 11.88%(-20.86%,43.72%) | 173.39(122.55,221.38) | 11.43%(-18.65%,42.09%) |
| Croatia | 2.16(1.66,2.74) | -48.71%(-61.04%, -32.69%) | 72.57(57.18,92.12) | -44.38%(-56.59%, -28.13%) |
| Czech Republic | 2.48(2.03,3.03) | -49.54%(-58.85%, -38.22%) | 83.01(68.18,100.26) | -47.43%(-56.58%, -36.27%) |
| Hungary | 3.20(2.61,3.94) | -48.62%(-57.95%, -37.17%) | 111.83(91.65,137.10) | -49.30%(-58.08%, -38.26%) |
| Macedonia | 3.20(2.38,4.22) | -10.65%(-33.38%,18.35%) | 106.75(79.14,137.11) | -15.48%(-35.70%,11.75%) |
| Montenegro | 2.72(2.22,3.44) | -5.56%(-31.93%,17.95%) | 99.22(81.46,120.29) | -5.55%(-27.83%,16.54%) |
| Poland | 3.48(2.74,4.36) | -47.20%(-58.00%, -33.67%) | 113.95(91.83,141.72) | -45.67%(-55.91%, -32.50%) |
| Romania | 6.26(4.56,7.76) | -12.81%(-37.86%,8.31%) | 223.93(163.04,277.39) | -13.62%(-33.38%,6.18%) |
| Serbia | 4.84(3.64,6.27) | -23.67%(-42.42%,1.26%) | 165.30(122.80,213.54) | -21.98%(-40.28%,4.23%) |
| Slovakia | 2.90(1.93,3.75) | -25.71%(-47.34%, -1.23%) | 100.32(69.52,128.77) | -25.72%(-46.22%, -1.51%) |
| Slovenia | 1.63(1.21,2.17) | -55.08%(-69.03%, -35.33%) | 56.30(43.51,73.89) | -52.07%(-66.49%, -32.87%) |

| | | | | |
|---|---|---|---|---|
| **Eastern Europe** | **8.25(6.92,9.43)** | **44.58%(24.34%,64.22%)** | **402.54(330.91,467.77)** | **106.62%(78.68%,131.89%)** |
| Belarus | 5.23(4.37,6.48) | -8.44%(-24.04%,13.72%) | 216.04(185.86,259.82) | 12.88%(-4.31%,34.25%) |
| Estonia | 4.37(3.65,5.23) | -30.83%(-42.69%, -16.68%) | 188.38(162.39,219.80) | -4.86%(-18.72%,13.58%) |
| Latvia | 5.60(4.91,6.56) | 1.97%(-12.29%,22.63%) | 236.84(212.28,273.25) | 35.68%(20.51%,57.84%) |
| Lithuania | 3.86(3.16,4.62) | -34.59%(-45.99%, -21.10%) | 134.42(111.81,160.52) | -29.73%(-41.08%, -15.91%) |
| Moldova | 6.42(5.73,7.26) | -2.54%(-13.40%,11.09%) | 285.28(256.60,318.19) | 25.81%(13.85%,41.26%) |
| Russian Federation | 8.28(6.76,9.68) | 67.29%(39.98%,90.71%) | 414.40(331.98,488.36) | 142.46%(102.24%,174.29%) |
| Ukraine | 9.33(7.90,10.96) | 22.88%(3.91%,89.89%) | 438.55(365.00,514.50) | 69.36%(45.92%,141.45%) |
| **North Africa and Middle East** | **2.44(1.91,3.26)** | **4.60%(-15.77%,47.22%)** | **97.39(74.92,139.08)** | **17.28%(-8.08%,66.20%)** |
| Afghanistan | 4.43(2.05,6.94) | -6.36%(-34.91%,44.85%) | 156.78(66.43,261.34) | -10.03%(-39.33%,49.88%) |
| Algeria | 2.53(1.68,4.76) | -34.69%(-55.58%,17.21%) | 92.71(60.86,195.95) | -32.63%(-54.06%,27.19%) |
| Bahrain | 1.41(1.12,1.84) | -44.74%(-57.34%, -23.95%) | 44.50(36.14,55.74) | -43.91%(-55.18%, -28.08%) |
| Egypt | 0.82(0.57,1.17) | -22.76%(-45.70%,6.27%) | 35.26(26.32,46.98) | -18.99%(-34.97%,0.55%) |
| Iran | 1.46(1.14,1.76) | -12.33%(-33.28%,13.78%) | 56.21(45.65,69.45) | -2.04%(-18.39%,23.51%) |
| Iraq | 1.35(0.98,1.79) | -13.20%(-39.63%,27.63%) | 53.41(39.23,70.81) | -10.90%(-35.17%,25.87%) |
| Jordan | 1.13(0.86,1.45) | -38.87%(-53.86%, -10.54%) | 42.69(33.59,53.71) | -32.74%(-47.20%, -8.96%) |
| Kuwait | 0.75(0.56,1.01) | -42.16%(-56.96%, -17.81%) | 29.53(22.87,39.78) | -35.19%(-48.27%, -16.12%) |
| Lebanon | 1.86(1.06,3.86) | -31.68%(-59.76%,35.01%) | 74.38(40.32,171.39) | -29.27%(-60.05%,44.27%) |
| Libya | 2.62(1.55,5.35) | 9.33%(-33.88%,134.34%) | 101.81(55.50,235.94) | 19.57%(-30.51%,182.69%) |

| | | | | |
|---|---|---|---|---|
| Morocco | 4.63(2.79,8.11) | -4.74%(-37.71%,70.83%) | 166.10(94.40,330.24) | -1.19%(-37.98%,90.89%) |
| Palestine | 1.71(1.08,2.06) | -22.10%(-43.55%,10.37%) | 56.52(42.47,68.45) | -20.02%(-38.46%,7.17%) |
| Oman | 3.15(2.33,4.22) | 37.01%(-11.10%,115.05%) | 116.23(83.65,158.83) | 58.59%(3.81%,140.56%) |
| Qatar | 1.27(0.98,1.65) | -49.45%(-62.30%, -30.36%) | 36.60(29.15,46.83) | -47.39%(-58.35%, -32.17%) |
| Saudi Arabia | 1.90(1.25,3.52) | 30.72%(-27.53%,150.70%) | 80.10(51.53,152.26) | 48.84%(-13.85%,188.35%) |
| Sudan | 11.90(7.05,21.04) | 309.78%(97.05%,807.76%) | 561.37(317.43,994.85) | 376.13%(110.97%,947.30%) |
| Syria | 0.97(0.74,1.29) | -22.20%(-43.77%,17.06%) | 40.02(30.94,52.51) | -20.23%(-38.68%,9.77%) |
| Tunisia | 2.08(1.12,5.13) | 13.04%(-39.34%,160.00%) | 87.33(45.55,231.53) | 24.94%(-34.18%,208.54%) |
| Turkey | 1.58(1.14,1.94) | -32.55%(-48.99%,4.31%) | 50.71(40.31,61.15) | -33.65%(-47.99%, -3.67%) |
| United Arab Emirates | 3.72(1.34,14.32) | -23.05%(-68.34%,215.48%) | 100.45(42.45,333.39) | -14.80%(-62.03%,187.14%) |
| Yemen | 3.07(1.43,7.66) | -1.09%(-53.59%,165.07%) | 121.14(54.82,337.34) | 7.48%(-56.34%,209.47%) |
| **Central Asia** | **4.70(4.19,5.33)** | **-21.65%(-29.35%, -10.90%)** | **172.71(153.01,197.65)** | **-15.40%(-23.14%, -5.57%)** |
| Armenia | 4.37(3.58,5.21) | -22.92%(-37.98%, -6.26%) | 153.89(128.19,184.77) | -21.45%(-35.09%, -5.30%) |
| Azerbaijan | 3.48(2.67,4.98) | -24.35%(-42.49%, -0.92%) | 121.15(94.08,162.20) | -25.94%(-42.56%, -5.10%) |
| Georgia | 4.55(3.66,5.47) | -25.96%(-41.00%, -8.68%) | 169.72(134.90,203.37) | -20.93%(-36.35%, -2.82%) |
| Kazakhstan | 4.95(4.25,5.86) | -31.18%(-41.18%, -15.12%) | 188.40(163.06,221.13) | -17.70%(-28.29%, -4.84%) |
| Kyrgyzstan | 5.75(4.79,6.65) | -24.10%(-35.00%, -11.58%) | 212.30(181.60,243.80) | -16.33%(-27.31%, -3.90%) |
| Mongolia | 7.17(5.19,9.63) | -29.10%(-47.96%, -1.41%) | 220.25(163.72,301.87) | -30.90%(-49.33%, -3.30%) |
| Tajikistan | 3.04(2.41,4.19) | -34.45%(-50.12%,5.37%) | 110.17(89.41,151.77) | -32.46%(-46.94%,0.46%) |

| | | | | |
|---|---|---|---|---|
| Turkmenistan | 4.59(3.49,5.78) | -17.12%(-34.67%,3.89%) | 183.66(142.73,229.10) | -11.37%(-29.01%,9.47%) |
| Uzbekistan | 5.15(4.20,6.14) | 4.35%(-15.17%,25.54%) | 187.40(153.69,223.25) | 5.64%(-13.14%,25.09%) |
| **South Asia** | **5.63(4.75,7.10)** | **1.35%(-17.53%,29.91%)** | **226.23(194.48,276.96)** | **17.54%(-3.87%,51.73%)** |
| Bangladesh | 3.24(1.96,4.76) | -55.45%(-69.36%, -23.16%) | 111.32(70.83,162.14) | -52.79%(-68.31%, -15.42%) |
| Bhutan | 6.76(2.73,23.76) | -15.31%(-63.81%,188.18%) | 272.33(94.54,1058,62) | -2.60%(-65.27%,270.50%) |
| India | 6.13(5.16,7.53) | 8.29%(-12.18%,39.20%) | 249.84(215.57,296.76) | 26.57%(4.36%,61.36%) |
| Nepal | 8.09(3.33,25.94) | 4.07%(-52.80%,264.87%) | 324.41(123.59,1072.81) | 16.98%(-52.79%,341.22%) |
| Pakistan | 3.15(1.93,6.82) | 23.08%(-25.89%,157.60%) | 123.17(71.90,284.29) | 30.40%(-24.92%,193.26%) |
| **Southeast Asia** | **8.14(7.10,9.92)** | **29.60%(7.10%,66.54%)** | **351.79(308.94,412.06)** | **60.39%(30.13%,107.11%)** |
| Cambodia | 11.91(9.10,15.64) | 39.77%(-0.13%,135.66%) | 501.00(378.94,661.17) | 68.48%(18.25%,185.47%) |
| Indonesia | 5.09(3.82,7.69) | -2.35%(-21.93%,30.31%) | 220.12(177.70,285.30) | 21.58%(-2.80%,65.15%) |
| Laos | 9.65(3.85,42.00) | 6.57%(-59.04%,540.01%) | 404.99(155.43,1890.97) | 27.94%(-54.89%,695.40%) |
| Malaysia | 6.30(4.82,7.76) | -7.68%(-27.59%,21.11%) | 227.39(181.49,278.72) | 9.64%(-13.26%,43.71%) |
| Maldives | 2.04(1.63,2.55) | -64.46%(-75.85%, -15.26%) | 66.88(54.19,83.99) | -66.90%(-77.22%, -26.26%) |
| Mauritius | 7.60(6.69,8.59) | 20.84%(5.05%,40.15%) | 327.80(288.82,364.92) | 62.93%(43.67%,86.12%) |
| Myanmar | 10.37(8.37,13.80) | 28.21%(-9.15%,105.08%) | 477.48(380.26,602.12) | 63.54%(12.10%,170.96%) |
| Philippines | 7.29(6.34,8.65) | 26.52%(3.87%,52.75%) | 343.06(303.12,392.93) | 56.85%(33.29%,86.36%) |
| Sri Lanka | 2.21(1.54,2.99) | -6.91%(-38.32%,33.70%) | 77.68(57.53,100.70) | -11.79%(-35.47%,19.00%) |
| Seychelles | 10.60(8.87,13.18) | -15.40%(-30.88%,3.11%) | 348.87(292.97,436.47) | -10.87%(-26.94%,9.33%) |

| | | | | |
|---|---|---|---|---|
| Thailand | 19.16(15.36,26.49) | 115.99%(64.87%,211.28%) | 874.41(700.62,1188.89) | 172.30%(109.28%,277.54%) |
| Timor-Leste | 19.33(4.40,95.24) | 174.44%(-35.40%,1109.11%) | 834.18(155.16,4211.48) | 225.50%(-34.16%,1240.19%) |
| Vietnam | 7.50(5.96,9.13) | 19.84%(-12.03%,67.47%) | 296.64(244.96,360.91) | 45.83%(8.99%,101.52%) |
| **East Asia** | **3.84(2.73,4.63)** | **14.06%(-40.31%,58.39%)** | **142.99(107.56,169.76)** | **28.48%(-29.65%,76.42%)** |
| China | 3.81(2.69,4.62) | 16.76%(-40.57%,63.83%) | 142.25(105.82,169.51) | 31.46%(-29.47%,83.03%) |
| North Korea | 6.05(3.56,13.16) | 6.18%(-37.96%,149.25%) | 229.09(126.05,573.21) | 19.48%(-37.28%,207.55%) |
| Taiwan(Province of China) | 3.03(2.42,3.98) | -51.41%(-61.70%, -35.89%) | 97.86(78.99,127.36) | -49.23%(-58.88%, -32.26%) |
| **Oceania** | **38.57(18.31,92.09)** | **314.64%(91.02%,878.79%)** | **1724.76(735.51,4282.37)** | **443.59%(120.71%,1259.71%)** |
| American Samoa | 5.81(4.38,7.67) | 13.31%(-20.81%,57.86%) | 202.66(148.88,277.02) | 23.47%(-14.54%,77.85%) |
| Federated States of Micronesia | 53.28(10.88,229.90) | 284.47%(-15.23%,1543.90%) | 2264.81(365.90,10140.15) | 384.02%(-10.78%,1859.98%) |
| Fiji | 12.71(5.91,16.78) | -10.90%(-38.13%,22.91%) | 408.17(207.54,538.39) | -11.31%(-36.53%,24.62%) |
| Guam | 6.32(4.31,9.91) | 24.85%(-20.53%,104.13%) | 268.31(174.50,438.74) | 65.04%(-1.06%,178.95%) |
| Kiribati | 41.03(30.42,52.08) | -12.59%(-33.84%,13.58%) | 1252.79(919.07,1603.69) | -17.05%(-38.93%,9.41%) |
| Marshall Islands | 16.54(7.02,58.44) | 32.45%(-36.41%,326.74%) | 647.82(249.86,2661.69) | 57.28%(-30.85%,445.42%) |
| Northern Mariana Islands | 8.95(6.87,11.38) | -5.37%(-32.08%,32.67%) | 296.95(219.43,388.90) | 0.67%(-30.54%,45.49%) |
| Papua New Guinea | 45.74(20.00,116.40) | 542.21%(148.23%,1561.00%) | 2070.62(792.98,5451.29) | 729.97%(184.01%,2165.94%) |
| Samoa | 12.56(4.76,55.75) | 56.91%(-36.27%,479.52%) | 517.08(174.15,2517.53) | 81.36%(-32.76%,617.33%) |
| Solomon Islands | 20.71(9.82,66.02) | 32.46%(-29.70%,268.75%) | 816.87(326.94,2801.87) | 39.85%(-31.04%,315.47%) |
| Tonga | 11.15(8.08,15.48) | -6.17%(-33.62%,34.50%) | 362.53(256.17,519.11) | -3.44%(-34.71%,43.38%) |

| | | | | |
|---|---|---|---|---|
| Vanuatu | 14.02(6.13,53.93) | 60.82%(-21.53%,382.79%) | 540.88(222.66,2279.47) | 79.09%(-18.41%,474.56%) |
| **High-income Asia Pacific** | **1.57(1.31,1.70)** | **-35.59%(-48.49, -29.97%)** | **60.93(52.14,70.49)** | **-25.04%(-39.08%, -18.69%)** |
| Brunei | 6.71(5.70,8.11) | -29.03%(-42.22%, -10.20%) | 211.07(177.49,256.39) | -26.83%(-41.39%, -5.77%) |
| Japan | 1.56(1.25,1.71) | -26.53%(-44.19%, -20.90%) | 61.04(49.19,70.60) | -10.37%(-32.04%, -3.24%) |
| Singapore | 1.77(1.58,2.00) | -60.57%(-64.57%, -54.98%) | 66.86(58.75,79.32) | -53.29%(-58.61%, -45.70%) |
| South Korea | 1.69(1.37,2.04) | -52.44%(-65.04%, -42.09%) | 61.38(51.45,74.89) | -51.31%(-59.84%, -41.46%) |
| **High-income North America** | **3.01(2.76,3.11)** | **-67.30%(-69.29%, -64.76%)** | **147.70(129.09,172.00)** | **-67.29%(-70.74%, -62.64%)** |
| Canada | 1.77(1.55,1.92) | -51.58%(-56.49%, -47.20%) | 82.93(71.22,98.62) | -49.21%(-54.76%, -42.80%) |
| Greenland | 6.00(4.97,7.34) | -47.69%(-58.46%, -33.65%) | 235.07(195.80,282.56) | -43.39%(-55.62%, -28.74%) |
| United States | 3.16(2.91,3.27) | -67.90%(-69.92%, -65.38%) | 155.17(135.60,180.95) | -67.92%(-71.42%, -63.25%) |
| **Western Europe** | **1.96(1.82,2.07)** | **-56.77%(-58.91%, -54.12%)** | **77.89(70.56,87.36)** | **-58.52%(-61.61%, -54.53%)** |
| Andorra | 4.41(2.03,12.34) | -1.64%(-52.52%,146.66%) | 169.90(67.54,504.10) | 0.21%(-57.47%,183.34%) |
| Austria | 1.71(1.55,2.08) | -61.65%(-65.32%, -49.42%) | 66.65(58.16,80.93) | -53.74%(-59.43%, -41.03%) |
| Belgium | 1.82(1.62,1.99) | -49.20%(-53.46%, -44.48%) | 71.84(62.82,82.41) | -40.70%(-46.98%, -33.46%) |
| Cyprus | 1.85(1.33,2.13) | -20.97%(-49.95%,9.51%) | 54.78(43.87,63.06) | -16.71%(-43.19%,8.65%) |
| Denmark | 2.06(1.86,2.69) | -57.03%(-61.52%, -35.52%) | 73.06(64.72,90.15) | -50.83%(-56.62%, -31.11%) |
| Finland | 1.05(0.79,1.17) | -39.56%(-52.90%, -30.96%) | 33.54(28.56,39.22) | -35.22%(-46.31%, -27.22%) |
| France | 2.05(1.85,2.24) | -71.59%(-73.98%, -69.10%) | 80.12(71.13,90.36) | -74.72%(-77.06%, -71.96%) |
| Germany | 1.88(1.72,2.04) | -57.43%(-60.73%, -53.87%) | 68.87(61.78,78.40) | -58.87%(-62.33%, -54.29%) |

| | | | | |
|---|---|---|---|---|
| Greece | 1.69(1.54,1.90) | -37.22%(-42.38%, -29.25%) | 56.76(51.15,65.00) | -32.85%(-38.51%, -23.75%) |
| Iceland | 1.10(0.93,1.27) | -56.02%(-62.13%, -46.19%) | 38.70(32.77,45.94) | -54.35%(-60.63%, -45.20%) |
| Ireland | 1.63(1.32,1.86) | -36.11%(-50.78%, -26.09%) | 60.95(47.21,70.74) | -32.69%(-45.51%, -22.13%) |
| Israel | 1.65(1.36,1.82) | -25.36%(-46.41%, -16.24%) | 63.14(53.10,73.05) | -18.46%(-37.09%, -6.92%) |
| Italy | 1.89(1.42,2.02) | -47.41%(-62.61%, -39.98%) | 78.10(63.46,89.35) | -56.58%(-65.86%, -48.98%) |
| Luxembourg | 1.30(1.14,1.57) | -62.18%(-67.15%, -54.85%) | 49.72(43.08,59.33) | -59.01%(-64.22%, -51.43%) |
| Malta | 1.18(1.02,1.38) | -50.82%(-57.44%, -42.08%) | 47.05(40.62,55.58) | -42.63%(-50.19%, -33.35%) |
| Netherlands | 1.43(1.27,1.56) | -59.47%(-62.74%, -56.02%) | 55.25(48.19,63.24) | -60.94%(-64.92%, -56.59%) |
| Norway | 1.72(1.55,1.89) | -48.27%(-51.68%, -44.00%) | 62.34(55.64,72.07) | -47.07%(-50.94%, -41.55%) |
| Portugal | 4.85(4.57,5.15) | -3.83%(-10.72%,4.69%) | 216.36(201.04,233.76) | 12.43%(3.60%,24.13%) |
| Spain | 2.23(1.83,2.38) | -62.89%(-68.05%, -58.62%) | 92.73(80.63,102.58) | -68.77%(-72.49%, -64.19%) |
| Sweden | 1.57(1.42,1.73) | -41.73%(-47.44%, -37.02%) | 53.20(47.38,61.45) | -41.35%(-47.54%, -35.65%) |
| Switzerland | 1.47(1.30,1.62) | -40.99%(-47.20%, -25.20%) | 61.24(52.82,73.30) | -25.11%(-33.81%, -6.98%) |
| United Kingdom | 1.83(1.72,2.27) | -52.90%(-55.03%, -41.16%) | 77.84(68.85,93.38) | -43.97%(-49.41%, -31.63%) |
| **Australasia** | **1.38(1.22,1.50)** | **-64.41%(-67.31%, -61.43%)** | **57.37(49.74,66.45)** | **-65.36%(-68.58%, -61.29%)** |
| Australia | 1.37(1.19,1.50) | -63.84%(-67.46%, -60.73%) | 57.02(49.13,66.25) | -65.37%(-68.81%, -61.49%) |
| New Zealand | 1.43(1.27,1.59) | -66.87%(-70.05%, -58.59%) | 59.62(51.72,69.79) | -65.12%(-69.06%, -55.55%) |

**Table 1.**

| Cause of death or DALYs | Deaths | | DALYs | |
|---|---|---|---|---|
| | 2019 age-standardized rate per 100,000 people | Percentage change in age-standardized rate,1990-2019 | 2019 age-standardized rate per 100,000 people | Percentage change in age-standardized rate,1990-2019 |
| **HIV/AIDS and sexually transmitted infections[a]** | 8.58(7.72,10.05) | 74.45% (43.34%,124.69%) | 463.58(408.90,548.55) | 71.89% (44.43%,110.60%) |
| **HIV/AIDS[b]** | 8.48(7.62,9.95) | 79.69% (45.78%,136.03%) | 447.44(394.82,533.10) | 78.98% (48.47%,124.52%) |
| **HIV/AIDS - Drug-susceptible Tuberculosis[c]** | 1.99(1.41,2.61) | 20.31% (-9.92%,74.02%) | 103.24(75.54, 134.47) | 18.24% (-9.92%,64.74%) |
| **HIV/AIDS - Multidrug-resistant Tuberculosis without extensive drug resistance[c]** | 0.19(0.08,0.36) | 2019.49% (1080.51%,3599.68%) | 9.57(4.10,17.39) | 1994.54% (1050.75%,3555.31%) |
| **HIV/AIDS - Extensively drug-resistant Tuberculosis[c]** | 0.01(0.00,0.01) | - | 0.32(0.14,0.59) | - |
| **HIV/AIDS resulting in other diseases[c]** | 6.30(5.26,7.80) | 105.86% (65.01%,166.11%) | 334.31(279.95,419.85) | 106.07% (69.16%,158.15%) |
| **Sexually transmitted infections excluding HIV[b]** | 0.10(0.08,0.11) | -49.73% (-55.97%, -40.92%) | 16.14(10.51,25.83) | -18.06% (-27.11%, -9.92%) |
| **Syphilis[c]** | 0.02(0.02,0.03) | -61.92% (-67.73%, -51.68%) | 1.24(0.95,1.51) | -51.20% (-58.69%, -39.42%) |

| | | | | |
|---|---|---|---|---|
| **Chlamydial infection[c]** | 0.01(0.01,0.01) | -40.98% (-49.35%, -30.94%) | 1.98(1.41,2.76) | -15.68% (-21.92%, -9.56%) |
| **Gonococcal infection[c]** | 0.04(0.03,0.04) | -43.19% (-50.85%, -34.58%) | 2.03(1.65,2.43) | -34.12% (-41.98%, -25.22%) |
| **Trichomoniasis[c]** | - | - | - | 4.89% (3.13%,6.55%) |
| **Genital herpes[c]** | - | - | - | 3.47% (2.04%,4.81%) |
| **Other sexually transmitted infections[c]** | 0.03(0.02,0.03) | -47.35% (-54.47%, -38.09%) | 4.28(3.09,5.78) | -20.47% (-26.66%, -14.25%) |
| **Neoplasms[a]** | 3.40(2.90,3.81) | -23.95% (-35.20%, -13.36%) | 107.20(90.52, 119.43) | -23.42% (-35.05%, -11.78%) |
| **Cervical cancer[b]** | 3.40(2.90,3.81) | -23.95% (-35.20%, -13.36%) | 107.20(90.52, 119.43) | -23.42% (-35.05%, -11.78%) |

[a]: cause groups at level 2

[b]: cause groups at level 3

[c]: cause groups at level 4
